# Perspective of the GEMSTONE Consortium on Current and Future Approaches to Functional Validation for Skeletal Genetic Disease Using Cellular, Molecular and Animal-Modeling Techniques

**DOI:** 10.3389/fendo.2021.731217

**Published:** 2021-11-30

**Authors:** Martina Rauner, Ines Foessl, Melissa M. Formosa, Erika Kague, Vid Prijatelj, Nerea Alonso Lopez, Bodhisattwa Banerjee, Dylan Bergen, Björn Busse, Ângelo Calado, Eleni Douni, Yankel Gabet, Natalia García Giralt, Daniel Grinberg, Nika M. Lovsin, Xavier Nogues Solan, Barbara Ostanek, Nathan J. Pavlos, Fernando Rivadeneira, Ivan Soldatovic, Jeroen van de Peppel, Bram van der Eerden, Wim van Hul, Susanna Balcells, Janja Marc, Sjur Reppe, Kent Søe, David Karasik

**Affiliations:** ^1^ Department of Medicine III, Faculty of Medicine, Technische Universität Dresden, Dresden, Germany; ^2^ University Hospital Carl Gustav Carus, Dresden, Germany; ^3^ Department of Internal Medicine, Division of Endocrinology and Diabetology, Endocrine Lab Platform, Medical University of Graz, Graz, Austria; ^4^ Department of Applied Biomedical Science, Faculty of Health Sciences, University of Malta, Msida, Malta; ^5^ Centre for Molecular Medicine and Biobanking, University of Malta, Msida, Malta; ^6^ School of Physiology, Pharmacology, and Neuroscience, Faculty of Life Sciences, University of Bristol, Bristol, United Kingdom; ^7^ Department of Oral and Maxillofacial Surgery, Erasmus MC, University Medical Center Rotterdam, Rotterdam, Netherlands; ^8^ Department of Internal Medicine, Erasmus MC, University Medical Center Rotterdam, Rotterdam, Netherlands; ^9^ The Generation R Study, Erasmus MC, University Medical Center Rotterdam, Rotterdam, Netherlands; ^10^ Rheumatology and Bone Disease Unit, CGEM, Institute of Genetics and Cancer (IGC), Edinburgh, United Kingdom; ^11^ Musculoskeletal Genetics Laboratory, Azrieli Faculty of Medicine, Bar-Ilan University, Safed, Israel; ^12^ Musculoskeletal Research Unit, Translational Health Sciences, Bristol Medical School, Faculty of Health Sciences, University of Bristol, Bristol, United Kingdom; ^13^ Department of Osteology and Biomechanics, University Medical Center Hamburg-Eppendorf, Hamburg, Germany; ^14^ Instituto de Medicina Molecular João Lobo Antunes, Faculdade de Medicina, Universidade de Lisboa, Centro Académico de Medicina de Lisboa, Lisbon, Portugal; ^15^ Department of Biotechnology, Agricultural University of Athens, Athens, Greece; ^16^ Institute for Bioinnovation, B.S.R.C. “Alexander Fleming”, Vari, Greece; ^17^ Department of Anatomy & Anthropology, Sackler Faculty of Medicine, Tel Aviv University, Tel Aviv, Israel; ^18^ Musculoskeletal Research Group, IMIM (Hospital del Mar Medical Research Institute), Centro de Investigación Biomédica en Red en Fragilidad y Envejecimiento Saludable (CIBERFES), ISCIII, Barcelona, Spain; ^19^ Department of Genetics, Microbiology and Statistics, Faculty of Biology, Universitat de Barcelona, CIBERER, IBUB, IRSJD, Barcelona, Spain; ^20^ Department of Clinical Biochemistry, Faculty of Pharmacy, University of Ljubljana, Ljubljana, Slovenia; ^21^ Bone Biology & Disease Laboratory, School of Biomedical Sciences, The University of Western Australia, Nedlands, WA, Australia; ^22^ Department of Internal Medicine, Erasmus MC, Rotterdam, Netherlands; ^23^ Institute of Medical Statistics and Informatic, Faculty of Medicine, University of Belgrade, Belgrade, Serbia; ^24^ Department of Medical Genetics, University of Antwerp, Antwerp, Belgium; ^25^ Unger-Vetlesen Institute, Lovisenberg Diaconal Hospital, Oslo, Norway; ^26^ Department of Plastic and Reconstructive Surgery, Oslo University Hospital, Oslo, Norway; ^27^ Department of Medical Biochemistry, Oslo University Hospital, Oslo, Norway; ^28^ Clinical Cell Biology, Department of Pathology, Odense University Hospital, Odense, Denmark; ^29^ Department of Clinical Research, University of Southern Denmark, Odense, Denmark; ^30^ Department of Molecular Medicine, University of Southern Denmark, Odense, Denmark; ^31^ Azrieli Faculty of Medicine, Bar-Ilan University, Ramat Gan, Israel; ^32^ Marcus Research Institute, Hebrew SeniorLife, Boston, MA, United States

**Keywords:** genome-wide association study, musculoskeletal disease, gene regulation, animal models, data integration analysis

## Abstract

The availability of large human datasets for genome-wide association studies (GWAS) and the advancement of sequencing technologies have boosted the identification of genetic variants in complex and rare diseases in the skeletal field. Yet, interpreting results from human association studies remains a challenge. To bridge the gap between genetic association and causality, a systematic functional investigation is necessary. Multiple unknowns exist for putative causal genes, including cellular localization of the molecular function. Intermediate traits (“endophenotypes”), e.g. molecular quantitative trait loci (molQTLs), are needed to identify mechanisms of underlying associations. Furthermore, index variants often reside in non-coding regions of the genome, therefore challenging for interpretation. Knowledge of non-coding variance (e.g. ncRNAs), repetitive sequences, and regulatory interactions between enhancers and their target genes is central for understanding causal genes in skeletal conditions. Animal models with deep skeletal phenotyping and cell culture models have already facilitated fine mapping of some association signals, elucidated gene mechanisms, and revealed disease-relevant biology. However, to accelerate research towards bridging the current gap between association and causality in skeletal diseases, alternative *in vivo* platforms need to be used and developed in parallel with the current -omics and traditional *in vivo* resources. Therefore, we argue that as a field we need to establish resource-sharing standards to collectively address complex research questions. These standards will promote data integration from various -omics technologies and functional dissection of human complex traits. In this mission statement, we review the current available resources and as a group propose a consensus to facilitate resource sharing using existing and future resources. Such coordination efforts will maximize the acquisition of knowledge from different approaches and thus reduce redundancy and duplication of resources. These measures will help to understand the pathogenesis of osteoporosis and other skeletal diseases towards defining new and more efficient therapeutic targets.

## Large GWAS Have Identified Multiple Loci That Are Associated With Complex Skeletal Traits

In the last decade, a series of large and well-powered studies have dramatically increased our appreciation of a multitude of genetic factors that influence skeletal diseases, including osteoporosis. Significant advances of the post-genomic era are expected to translate into enhanced ability to predict who is at risk for disease, and to enable better treatment of those who already have bone disease (1, 2). GWAS and whole genome sequencing (WGS) analyses have transformed the genetic analysis of complex diseases in general and osteoporosis in particular. The results of GWAS are increasingly being used by the pharmaceutical industry as an effective means of prioritizing compounds for development, as well as for repurposing existing medications for new indications (3).

Bone mineral density (BMD) remains the strongest predictor of fracture risk. As BMD also is highly heritable, it is frequently used in GWAS (4). The most significant study to date on the genetics of osteoporosis is a 2019 UK Biobank study involving approximately 420,000 participants (5). This study identified a total of 518 loci associated with estimated heel BMD, of which 301 were *new* loci. Of note, GWAS is mostly useful to identify common variants (usually defined as variants with a minor allele frequency >1%). On the other hand, genetic mutations are frequently discovered for less-common skeletal diseases; these mutations might be rare. A recent strategy that has already been employed in skeletal research is to use WGS, which is able to identify rare variants with large effect sizes. Such studies have identified several rare mutations in *LGR4* (6) and *COL1A2* (7) loci that are associated with low BMD. One particularly powerful study combining sequencing and GWAS identified a non-coding variant at *EN1* (minor allele frequency = 1.6%) that also has large effects on BMD (8).

Interpreting results from human association studies remains a challenge, especially nominating causal genes for complex traits based on genome-wide significant SNPs (9). To bridge the gap between the genetic association and molecular function, a systematic functional investigation is necessary to interpret GWAS variants and to infer the exact disease-causing genes, or genes they regulate, and the cells in which they act (9). Here we review current practice for functional dissection of human complex traits and propose a roadmap for data integration and target prioritization for the skeletal outcomes.

## Causality of Genetic Mutations Associated With Rare Skeletal Diseases Requires Proof

Rare skeletal disorders span a broad clinical spectrum of bone-related pathologies, occasionally exhibiting extra-skeletal manifestations. Besides being genetically heterogeneous, the severity of these disorders is highly variable, ranging from neonatal lethality to minor complications discovered incidentally during adulthood (10, 11).

In contrast to complex traits having a multifactorial genetic etiology, genetic studies of monogenic diseases focus primarily on identifying the underlying causal rare variant(s) in affected patients, isolated or as members of a multiplex family (12). The first step consists of deep phenotyping of the clinical skeletal manifestations. The differential diagnosis based on the clinical and radiological observations might strongly indicate one candidate gene as an initial hypothesis explaining the underlying genetic causality. However, most cases have an uncertain genetic basis; necessitating hypothesis-free approaches. These include on the one hand homozygosity mapping and linkage analysis in multiplex families resulting in the delineation of a genomic region where the disease-causing gene resides. On the other hand, and potentially in combination with the previous approaches, high-throughput sequencing provides insight into the genetic variation within an individual. The widespread availability of recent -omics technologies has permitted researchers to focus their efforts on this approach by utilizing customized gene panels, whole exome sequencing (WES) or WGS. Nonetheless, all genetic discoveries resulting from traditional approaches such as linkage analysis or high-throughput technologies require translational assessment and annotation using *in vitro* or *ex vivo* bone cell work and/or *in vivo* knockout models to confirm disease association.

**Figure 1:**
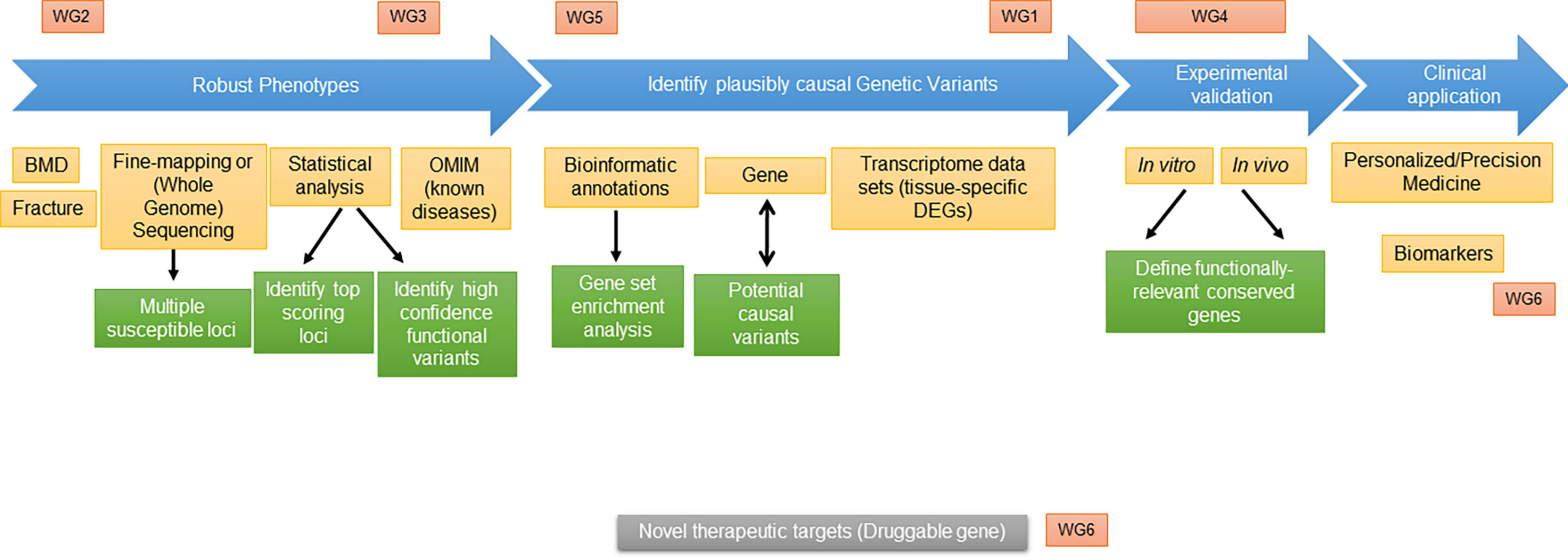
Scheme of proposed “roadmap” and integration of GEMSTONE Working Groups.

*Examples of successful genetic findings with functional validation* (i). Four consanguineous and distantly related individuals with autosomal recessive osteopetrosis were analyzed using homozygosity mapping (13). A single 1.22 Mb genomic region shared by all affected subjects was identified on chromosome 7, harboring five genes: *NFE2L3*, *HNRNPA2B1*, *CBX3*, *SNX10*, and *SKAP2*. Among these genes, *SNX10* (sorting nexin 10) readily stands out as an excellent candidate due to its involvement in endosome homeostasis. A missense mutation was identified in all affected patients and was hence taken forward for functional investigation, whereby osteoclasts derived from monocytes of patients revealed gross abnormalities in the endocytic system and resorptive activity, abnormal *SNX10* expression and altered subcellular localization of the encoded protein. Subsequently, *Snx10* silencing experiments in mice highlighted the essential role of SNX10 in osteoclast vesicle trafficking and osteoclastic bone resorption (14, 15).

(ii) WES was conducted on three sisters. These sisters had a history of atypical femoral fractures after long-term bisphosphonate treatment for their underlying osteoporosis (16). WES analyses identified the presence of a rare missense mutation in *GGPS1* (Geranylgeranyl Diphosphate Synthase 1), encoding the GGPPS enzyme, which acts downstream of the point of bisphosphonate action. Functional validation of the exact missense change, together with gene knockdown in osteoblasts and osteoclasts, was essential to confirm causality and to demonstrate the importance of the gene in atypical femoral fracture susceptibility (17). Additionally, other WES-prioritized variants, such as *CYP1A1*, were found mutated in other atypical femoral fracture cases (17, 18), opening the possibility of digenic or oligogenic inheritance. It might also reflect the idea that clinical variability, observed in many monogenic diseases, can be explained by variants in modifier genes (19). The discovery of such genetic variants opens an application window into personalized medicine (20).

(iii) Well known in the field, is the G171V missense variant in the gene encoding *LRP5*. The discovery of this variant was the outcome of a linkage study combined with a focused sequencing effort in a large family with several cases characterized by high bone mass (21). A combination of genetic and functional studies soon provided strong support for the involvement of *LRP5* (22). Loss-of-function mutations in *LRP5* explain the low bone mass in osteoporosis-pseudoglioma syndrome and other missense mutations in the same domain were identified in high bone mass phenotypes (23, 24). Soon afterwards, a wealth of *in vitro* and *in vivo* data confirmed the important role of the *LRP5* gene in the regulation of bone mass (25), corroborated by GWAS and candidate gene association studies indicating the effect of a few common *LRP5* variants on BMD and the risk for osteoporosis in the general population (26). Indeed, *LRP*5 is an empirical example of a gene that may harbor mutations or polymorphisms contributing to monogenic or complex forms of abnormal bone mass respectively. Identification of the defective gene in monogenic diseases serves as an optimal natural-occurring ‘knockout’ model, with population-based studies enabling gene prioritization and validation, disentangling the underlying pathogenesis in monogenic conditions. Other examples of genes and loci exist, discussed in more detail in this issue’s paper (12). Monogenic diseases are also not only constrained to mutations in the protein-coding regions. It has been shown the homozygous 52-kb deletion in the SOST-MEOX1 intergenic region on 17q12-q21 occurs in van Buchem disease patients (27). This region 35 kb downstream of the *SOST* gene fosters a long-range enhancer for it. Thus, the patients have reduced *SOST* transcription which reflects in lower sclerostin levels (28) (**Figure 1**).

*Benefits from recent advances in sequencing technologies:* Novel techniques have remarkably facilitated the elucidation of the underlying molecular etiology of many rare skeletal diseases. Indeed, this has enabled the classification of conditions based on the implicated genetic defect and/or the altered metabolic/signaling pathways. This is why, the monogenic mutations can serve as human knockout models and help to uncover the gene function while GWAS findings serve to prioritize genes to scrutinize the cause of the monogenic conditions (12). In the case of osteogenesis imperfecta (OI), genetic discoveries have prompted a nosology revision of the existing classification, with causative genes added as new subtypes of the OI types I-V (29). A total of 17 genetic causes of OI have been described to date, with inheritance patterns ranging from autosomal dominant (e.g. *COL1A1, COL1A2, IFITM5*), autosomal recessive (e.g. *LEPRE1, PPIB, SERPINH1, PLOD2, BMP1, WNT1*), and X-linked (*PLS3*). An impressive 92% of 461 skeletal disorders described by the Nosology Committee of the International Skeletal Dysplasia Society have been solved at the genetic level thanks to high-throughput sequencing, creating new well-defined entities and sub-classifications of previously unknown or ill-defined skeletal disorders (11). Improved nosology based on careful clinical phenotyping coupled with genetic data leads to better patient care, both in terms of diagnosis and treatment (30). The discovery of causative genes and defective proteins has aided in the diagnosis, prognosis, and management of affected individuals, accelerating the development of personalized therapy. A good example is the treatment-option of bone marrow transplantation in patients with malignant recessive osteopetrosis. Unraveling the genetic cause in these patients before treatment decision is essential, as in RANKL deficiency, bone marrow transplantation will not have any beneficial effect (31–33). Finally, the identification of genes involved in monogenic diseases have resulted in novel treatments for osteoporosis, as is the case for denosumab, an anti-RANKL monoclonal antibody (34, 35) and romosozumab, an antibody against the Wnt-inhibitor sclerostin (36).

## Need for Intermediate Traits (Endophenotypes) and New Biomarkers of Skeletal Disease

Fragility fractures represent a very complex phenotype. So far, most genetic studies have focused on BMD rather than bone fracture risk. There is a realization that the genes affecting BMD are not necessarily the same genes that influence fracture risk (37); however, there are no such indications from the fracture GWAS (38). There is the need for new phenotypes, which will enable and support the causal genes validation.

Imaging techniques like QCT, high resolution peripheral QCT, magnetic resonance imaging (MRI), and radiofrequency echographic multi spectrometry (39), together with fracture and BMD traits are considered as measurable “exophenotypes”, while “endophenotypes” - parameters that are more biologically proximal to gene actions - are currently lacking. Here we define the term, endophenotype (a.k.a. intermediate phenotype), as a characteristic able to mark genetic vulnerability independent of the clinical state (40, 41). Therefore, endophenotypes have the potential to identify the genetic dysfunction prior to disease manifestation. Similar to exophenotypes, the endophenotypes might be influenced by many genes, each with a relatively small effect, making endophenotype-identification difficult. Lifestyle factors (e.g. diet, physical activity) as well as other confounders can influence the exophenotypes such as BMD, QCT, fractures and others, and can mask the effects of genetic factors that we aim to assess in functional genomics (42). Hence, the main advantage of endophenotypes *vs*. exophenotypes is that their correlation with genetic changes is stronger, as they are more proximal to genes. The levels of molecules like proteins, metabolites, miRNA in bone cells, bone tissue, and/or in the blood can be chosen as the endophenotype.

*Current status and needs in the field:* It is desirable to have endophenotypes that can be used as specific biomarkers of bone cell activities in order to compensate for the shortcomings of BMD. In contrast to BMD, the potential serum/plasma bone biomarkers would ideally be able to reflect bone remodeling (43–46) in a dynamic fashion. Increased bone turnover results in microarchitectural deterioration of bone and has been associated with fracture risk independent of BMD. However, the evidence is currently not robust enough to use any biomarker in the fracture risk prediction tool (47).

Examples of established bone formation biomarkers used as endophenotypes in treatment monitoring are procollagen I N-terminal propeptides (PINP), bone-specific alkaline phosphatase, procollagen type I C-terminal propeptide (PICP) and osteocalcin, while C-terminal telopeptide of type I collagen (CTX), N-terminal telopeptide of type I collagen (NTX), tartrate-resistant acid phosphatase isoenzyme 5b, C-terminal crosslinked telopeptide of type I collagen, (ICTP), and deoxy-pyridinoline serve as resorption biomarkers (48). Bone biomarkers’ specificity for their respective process is convincing (48). However, there are major challenges even with the recommended reference markers CTX and PINP. Namely, especially CTX fluctuates during the day requiring blood samples to be collected from fasting patients in the morning, and both CTX and PINP vary tremendously among different individuals (49). Therefore, new biomarkers are being investigated, including proteins regulating bone resorption (RANKL, OPG), bone formation [sclerostin (50)] or bone non-collagenous proteins [periostin (51)].

*miRNAs as endophenotype markers:* Examples of potential new molecular biomarkers include non-coding RNAs (ncRNAs), of which miRNAs currently seem to be more promising (52, 53). They are small, 20–24 nucleotides long, noncoding, single-stranded RNA molecules that act as post-transcriptional regulators of gene expression. A cluster of miRNAs can target a single gene, and every single miRNA can regulate several different protein-coding genes. Their role in bone homeostasis is well established since miRNAs were shown to significantly affect the differentiation, proliferation, and function of both osteoblasts and osteoclasts (54, 55). Besides being intracellular, they are also present in several biological fluids where they are remarkably stable. Several studies have shown differences in circulating miRNA levels between osteoporotic and control subjects, both in primary and secondary osteoporosis [reviewed in (56)]. Based on these studies, it was proposed that circulating miRNAs could serve as a clinical tool for fracture risk prediction giving additional information on bone metabolism not captured by BMD, FRAX^®^, or traditional bone turnover markers. A commercial test for fracture risk based on a panel of 19 miRNAs called OsteomiR™ was shown to effectively assess fracture risk (52). A cost utility model showed that its implementation could reduce fracture incidence compared with standard approaches such as monitoring BMD, no monitoring, or FRAX^®^ calculation alone (57). However, the key circulating miRNAs in osteoporosis are not consistent between studies, and before their implementation in routine clinical practice can become a reality, further studies are required to obtain validated disease-specific signatures (58, 59).

*Endophenotypes as functional markers:* Described endophenotypes are also relevant for *in vitro* functional validation of GWAS hits. Ideally, the endophenotypes should be able to reflect the “effects” of a particular genetic variation even with subtle changes in gene expression i.e. mRNA, protein level/activity, or other metabolites levels. In this context, identification and selection of endophenotypes depends on the position of the genetic variation of interest. If it is positioned in regulatory gene regions (e.g. the promoter), the gene expression and mRNA level is a well suited endophenotype. On the other hand, in the case of coding genetic variants, the protein should be qualitatively and quantitatively analyzed. Suggested approaches to functionally evaluate SNPs are presented in **Table 1**.

**Table 1:** Approaches in the functional evaluation of SNPs.

Computational analyses	Outcome
expression quantitative trait locus (eQTL)	SNPs regulating gene expression
allele specific expression quantitative trait locus (aseQTL)	allele-specific expression
regulatory trait concordance (RTC), joint likelihood mapping (JLIM)	shared causal variants between eQTL and a trait (e.g. BMD)
functional annotation (Combined Annotation Dependent Depletion (CADD), Eigen, RegulomeDB, LINSIGHT, GWAVA)	the most probable functional SNPs
**Functional assays**	**Outcome**
high‐throughput chromosome conformation capture (Hi‐C)	Whole-genome chromatin interaction
dual luciferase assays	validation of allele-specific promoter or enhancer activity
CRISPR/Cas9, dCas9-KRAB, dCas9-DNA demethylase	direct evidence of long-range regulation
ChIP, RNAi, Cotransfection assays	TF binding affinity of allele-specific enhancer or promoter activity
animal models (knock-in, knock-out)	functional relevance of target gene to bone metabolism

**Figure 2:**
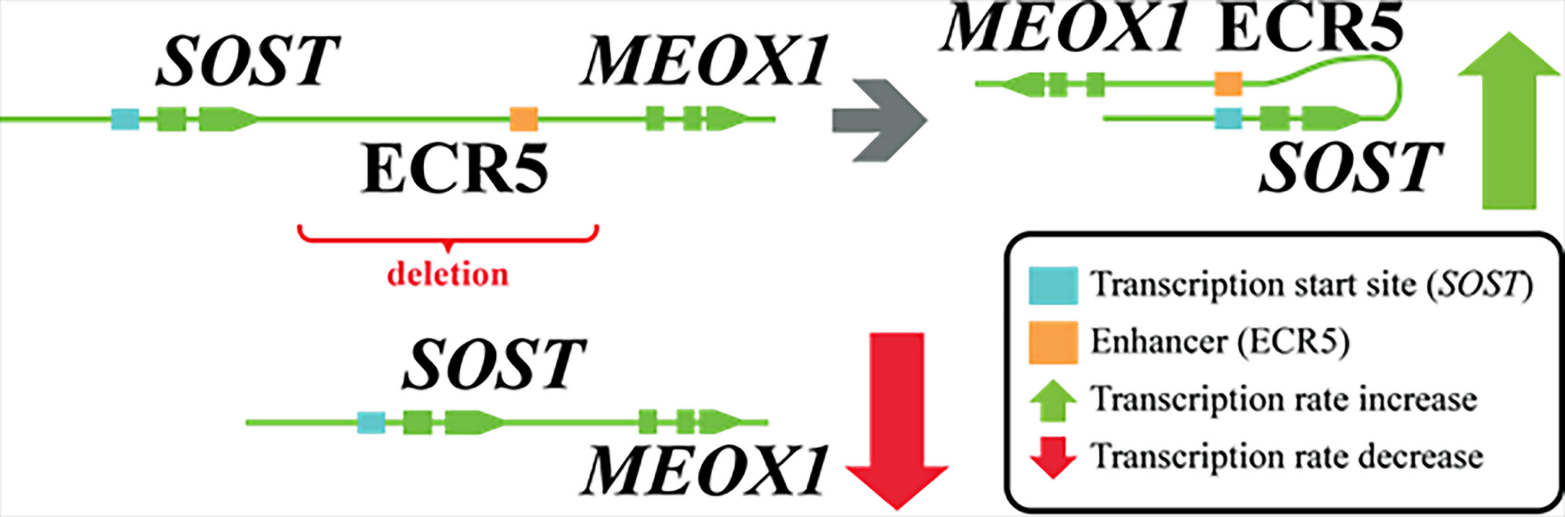
Genomic deletion affecting ECR5 enhancer for SOST and its effect.

In conclusion, endophenotypes that are needed for identification and evaluation of risk genes are important not just for progress in functional genomic research, but also for better prognosis and prevention of bone disease, which is ultimately the goal of the GEMSTONE consortium (**Figure 2**).

## Current Practice: Genomic Annotation and Establishing Causality

With the boom of genetic testing, an opportunity for establishing possible causalities for the skeletal traits and diseases based on genomics was discovered. A seminal paper published in 2003 laid out the foundations and conveyed the key message of the method called “Mendelian randomization” (60). The main focus of this method is to establish the relationship between an exposure (a SNP) and an outcome (an endo- or exo-phenotype) *via* a “proxy”, an instrumental variable, that within a specific degree of certainty cannot be influenced by neither intrinsic nor extrinsic influences, i.e. confounders. Thus, the association between the exposure and outcome can indeed be established to arise from the two alone and not due to other factors. The results of such analyses may predict both the direction as well as the effect-magnitude. In terms of genomics, SNPs were proven to be a good fit for these instrumental variables. With time and technological advancements, large-scale GWAS provided well-powered and reproducible association results for risk prediction of common diseases (61), including the skeletal field (62). Many such studies in the field of skeletal diseases have since been performed, with a notable one scrutinizing the clinical risk factors of fracture (38). In contrast to low BMD, an established “causal” determinant of fracture, three is no evidence to suggest that increasing vitamin D (25-hydroxyvitamin D) levels in “sufficient” individuals will modify fracture risk.

*Use of GWAS to identify quantitative trait loci (QTLs):* The method of Mendelian randomization is not constrained to the results of GWAS alone. SNPs can also act as QTLs where they correlate with the expression of genes (eQTLs). Other QTLs include protein expression (pQTLs) (63, 64), DNA methylation (mQTLs) (65, 66), and chromatin acetylation and chromatin accessibility QTLs [reviewed by (9)]. eQTLs are abundant, with 48% of common genetic variants estimated to act as eQTLs for at least one gene (9).

Within such Mendelian randomization configuration, the effect of gene expression, as an exposure, can be tested for association against chosen traits, the outcome(s). The challenge that such studies face is that gene expression is highly tissue-specific; thus, expression in one tissue may not fully predict expression in another. This is especially important since it is unclear which cells are the true drivers of a disease (i.e., in which cell type GWAS variants act), as the pathophysiology of complex diseases often implicates interactions of multiple cell types (9). As of yet, there are not many studies that implement gene expression from bulk bone tissue. There are some that leveraged eQTL data obtained from whole blood and tested for effect on estimated BMD (eBMD) (5), and more recently eQTLs obtained from osteoclast-like cells derived from human peripheral blood mononuclear cells were tested for effect on the same eBMD trait (67, 68) as well as in the case of the newly reported osteomorphs (69) using a mouse model.

*Co-localization:* Co-localization analyses integrate eQTL and GWAS data (70, 71). Within their scope, the position of the (usually) topmost associated SNP(s) on each locus between the two datasets are analyzed, with results indicating whether the same SNP(s) drive both the gene expression at that particular region and the GWAS signal (e.g. where the SNPs effect on the GWAS trait is mediated by the gene expression). The difference with the Mendelian randomization-based approach is that the co-localization does not estimate the effect size and direction, but provides the probability of (a) shared causal variant(s).

## Current Practice: Genomic Annotation for Coding and Non-Coding Regions

Identification of candidate genes is more straightforward for coding variants, which may directly disrupt the structure of a protein (9). However, as early as 2012, it was realized that only a minority of GWAS hits fall within transcribed regions, with most of them mapping to introns (4.9% and 41.2% respectively (72). The leftover majority of GWAS hits thus cannot be easily linked to a candidate causal gene.

**Figure 3:**
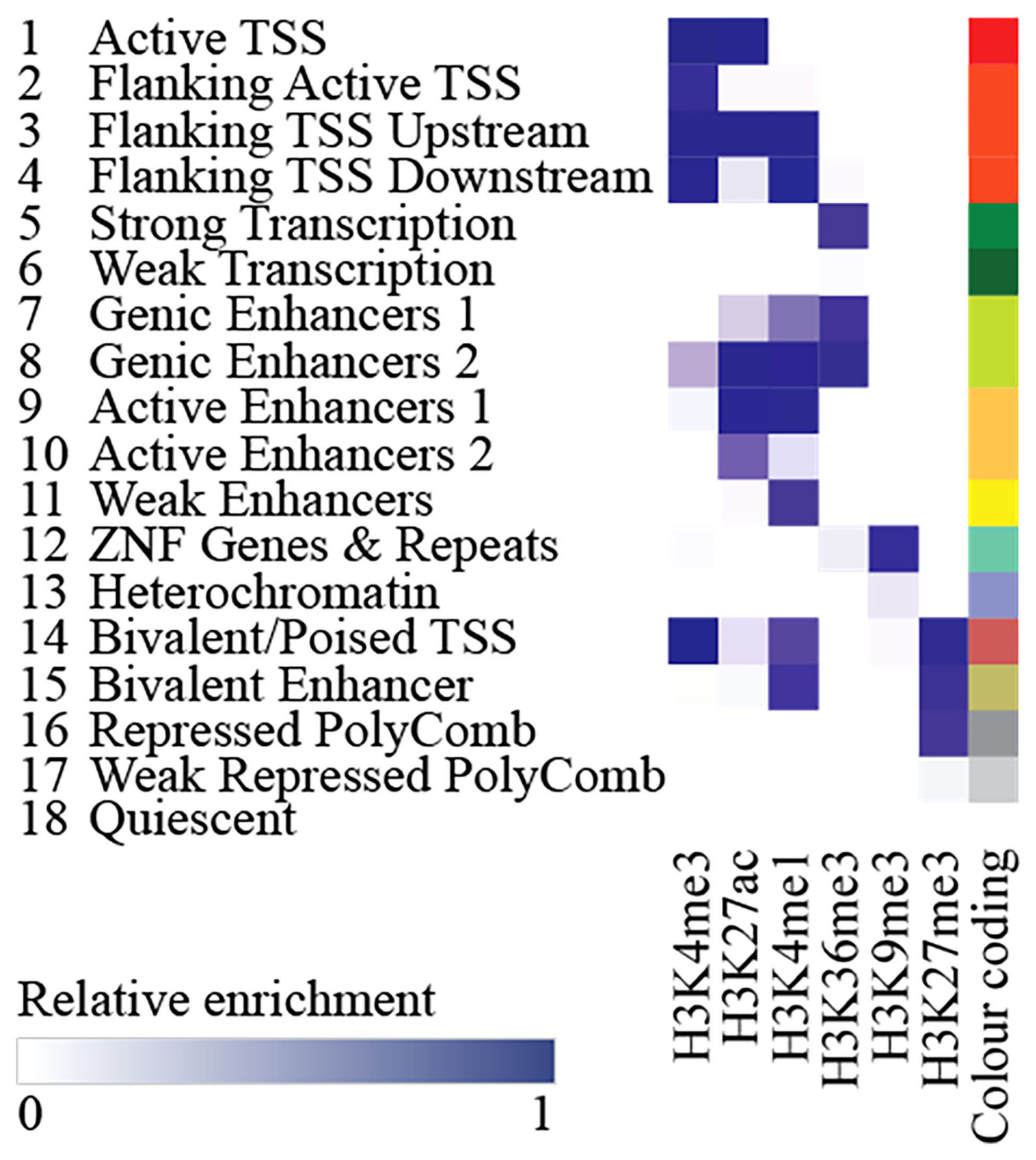
Chromatin state definitions in the 18-state chromatin model as defined by the relative enrichment of respective histone marks.

Also, in 2012, the ENCODE Project Consortium set out to map and describe functional elements encoded in the human genome across 1,640 data sets involving 147 different cell types, amongst which were also human osteoblasts (73). The mapping was expanded in 2015 by the Roadmap Epigenomics Consortium (74). The consortia assayed the available cells for eight histone modifications. By further integrating five specific histone modification marks (histone H3 lysine 4 trimethylation and monomethylation – H3K4me3 and H3K4me1 respectively; trimethylations of histone H3 lysine 36 – H3K36me3; histone H3 lysine 27 – H3K27me3; histone H3 lysine 9 – H3K9me3) they were able to build the 15-chromatin-state model (and later expand it to the 18-state model by inclusion of the histone H3 lysine 27 acetylation; **Figure 3**).

In short, by combining information on the methylation and acetylation dynamics, they were able to predict local chromatin states that were roughly divided into 8 active and 7 repressed states, now used to study the relationship between histone modification patterns, RNA expression levels, DNA methylation, and DNA accessibility. Their findings additionally showed tissue-specific enhancer regions, epigenomic dynamics during lineage specification, and both similarities and differences thereof between various tissue and cell types (74). Importantly, both consortia freely released their data repositories for use by others. This data can be integrated and tested against for enrichment e.g., by incorporating it into GARFIELD (GWAS Analysis of Regulatory of Functional Information Enrichment with LD correction) or by using the partitioned heritability function of the LDSC (linkage disequilibrium score) regression method (75, 76). This allows researchers to further functionally analyze and annotate their results and potentially discover novel cell and tissue specific genomic patterns.

One of the goals of *in silico* and *in vitro* methods is to translate findings to *in vivo* models. Historically, mouse – and more recently, zebrafish models - have been used to explore variants present in protein-coding regions. Recently it has been shown that despite poor evolutionary conservation in non-coding genome sequences, this model can still be used to compare enhancer activity of putative variants as predicted by *in silico* findings (77).

With WES being increasingly applied to large population-based settings, the American College of Medical Genetics and Genomics and the Association for Molecular Pathology (ACMG-AMP) have released standards and guidelines for the interpretation of sequence variants (78). According to these guidelines, the variants are classified as 1) benign, 2) likely benign, 3) uncertain significance, 4) likely/expected pathogenic, and 5) (known) highly pathogenic. Databases can follow this classification system, whereas others, such as Human Gene Mutation Database (HGMD), use their adaptation of functional classifications (79, 80). The Combined Annotation Dependent Depletion (CADD) (81) is a tool that uses a machine learning approach for scoring the deleteriousness of both coding as well as non-coding variants. It has been shown that the occurrence of known pathogenic and expected pathogenic variants in healthy populations are higher than expected based on disease prevalence (82–84). Many variants predicted to be potentially pathogenic have a lower than expected penetrance in healthy populations. To this effect, the UK BioBank has recently forayed into the venture of sequencing whole genomes of its participants, identifying rare variants to improve the prediction of monogenic and complex traits. The first tranche of results (n=49,960) was released to be used by the wider scientific community (85), with an addition of exome sequencing data on 150,000 volunteers added to the UK BioBank database (https://www.ukbiobank.ac.uk/2020/10/uk-biobank-makes-available-new-exome-sequencing-data).

As a proof of this concept, evaluation of WES data with clinical information has been done in a deeply phenotyped cohort study (86). They discovered 26 variant carriers, but only three of them experienced a clinical event related to the identified variant. When they consulted two main databases, ClinVar and HGMD, for clinical interpretation of the variants, they found a high degree of disagreement between the two databases. Moreover, the clinical classifications within ClinVar in different releases over five years (2014-2018) evidenced a trend of changing the clinical interpretation of formerly expected pathogenic variants towards class 1 (benign), 2 (likely benign) or 3 (uncertain significance). As shown, the definition of known pathogenic variants is ambiguous between databases, yet also differs between different versions of the same database. Moreover, potentially pathogenic variants do not always have a clinical impact. This presents challenges that researchers, as well as clinicians, face and must address while interpreting their findings.

## Non-coding variance and regulome

### ncRNAs/miRNAs

Recent technical advances in the high-throughput genomic platforms have revealed that only 1–2% of the human genome is protein-coding. Previous findings (72) suggest that GWAS variants could modify the regulatory activity of non-coding elements in a cell-type specific manner. Schmidt et al. (87) confirmed that GWAS SNPs are generally enriched in active regulatory regions compared to random SNPs. A vast majority of intergenic signals are represented by ncRNAs, thus implicating their potential role in contributing to the GWAS phenotype. The two most abundant types of regulatory ncRNAs are the miRNAs (~22 nucleotides) and long non-coding RNAs (lncRNAs, ≥200 nucleotides). To our knowledge, while a broad spectrum of ncRNAs has a potential impact on MSK metabolism, miRNAs have been investigated in bone diseases more than others. Most studies focused on measuring the levels of miRNAs in either bone tissue or in circulation to find disease specific miRNA signatures which could be used as biomarkers or endophenotypes and are described in the above section *“Need for Intermediate traits (endophenotypes) and new biomarkers of skeletal disease”.* Much less is so far known regarding genetic contribution to miRNA regulation.

Genetic contribution to circulating miRNA profiles has so far been demonstrated in monogenic types of osteoporosis caused by *WNT1* (53) or *PLS3* (88) mutations. Also, in a limited number of studies, SNPs affecting miRNA regulation have already been shown to contribute to the understanding of the genetic determinants of osteoporosis. These include polymorphisms in miRNA genes (miR-SNPs) and miRNA binding sites of target mRNAs (miR-TS-SNPs). The miR-SNPs can affect either miRNA’s transcription, its processing, or mRNA binding (89). Polymorphisms at or near miRNA target sites within a mRNA (miR-TS-SNPs) can either create or eliminate a miRNA binding site (54). A relevant miR-SNP found by GWAS is rs11614913, located in precursor *MIR196A2*, which was significantly associated with femoral neck and lumbar spine BMD (90), as well as with lumbar spine area derived from DXA scans and hip fractures (91). The variant was proposed to affect the stability of miR-196a-2 (90) and was experimentally confirmed to directly influence repression of hsa-miR-196a-5p target genes (91). An example of a functional miR-TS-SNPs is rs1048201 in basic fibroblast growth factor (FGF2) 3′ UTR which was associated with lumbar spine BMD and affected binding of hsa-miR-196a-3p, the other mature miRNA derived from previously mentioned MIR196A2 (92).

### ncRNAs/lncRNAs

Despite previously considered as “transcriptional noise,” lncRNAs are emerging as key regulators of major biological processes influencing development, differentiation, and disease (93–95). They are best known for assembling transcriptional machinery to trigger the initiation of transcription, recruiting epigenetic factors to modify chromatin state (94, 96). Some of the lncRNAs act as sponges for miRNAs, titrating them away from their target mRNAs (97, 98). In contrast, others are generated from antisense strands of coding genes and can directly modulate the coding gene translation *via* base pairing with the complementary mRNA (99).

The GWAS-associated variants may affect regulatory elements that modulate mRNA transcription level modifiers. For example, enhancers are context-specific; their current annotations are incomplete. Finucane et al. (76) showed that variants within enhancers specific to disease-relevant cell types explained a substantial proportion of heritability. Therefore, information must be integrated across tissue contexts and data sources to identify variants affecting enhancer function (95). Many eQTLs affect lncRNAs, which in turn can regulate protein-coding gene expression (100).

A tendency to assign lead variants preferentially to coding genes close to GWAS hits contributed to a disregard of the role of non-coding elements (101). We postulate that the upsurge of databases integrating SNPs and non-coding RNAs with novel technologies will facilitate the discovery of causal non-coding variants associated with skeletal phenotypes. The integration of these comprehensive datasets using a read-across framework will aid in prioritization and functional validation of candidates (102). Another problem affecting the ncRNAs, relevant for both lncRNAs and circular RNAs (circRNAs), is the paucity of targeted assays. Most of the expression data of skeletal tissues or cells available in the databases are microarray data, where only known protein-coding genes are included. For an exhaustive characterization of the expression pattern of both coding and non-coding genes, whole transcriptome sequencing should be performed with ribosomal RNA-depleted total RNA libraries instead of frequently used poly-A+ RNA-seq libraries.

## Repetitive Sequences

Traditional GWAS using microchip analysis has been limited to less than half of the genome, since the major part consists of various repeated sequences and retrotransposons in which accurate localization of genomic variants is not possible. This may, however, change with a trend towards WGS, enabling very long reads spanning repeated sequences with the newest DNA sequencing tools. The most abundant retrotransposon, Long Interspersed Nuclear Element 1 (*LINE1, L1*), is 6 kb long and constitutes 20% of the genome (103). Of relevance to bone metabolism, a recent study showed that blocking L1 activity hinders differentiation of bone marrow mesenchymal stromal cells (BMSC) into osteoblasts, while transfection of BMSC from osteoporotic women with the *L1* transcript stimulates osteoblast differentiation and bone production. In line with these results, the *L1* copy number was increased in bone from healthy postmenopausal women as compared to osteoporotic women {Mangiavacchi, 2019 #1773}.

A decrease in CpG methylation status of repetitive sequences has been demonstrated during osteogenic differentiation of BMSCs suggesting that their methylation status is important for the induction of osteogenic differentiation (104). In the blood of postmenopausal osteoporotic women, the methylation status of Alu short intersperse elements (SINEs) has been associated with lower BMD, suggesting a positive correlation between Alu hypomethylation and age-related phenotype such as loss of BMD (105). In rare cases, when retrotransposition occurs in germ cells, unhindered by the host restriction mechanisms, novel mutations arise in the host genome that can be vertically transmitted to the next generation. Mutations induced by repetitive sequences were also reported in genes involved in musculoskeletal development. Mouse mutant *chagun* with skeletal dysplasia and male infertility has been demonstrated to have LINE-1 insertion in *Poc1a* gene (106) [reviewed in (107)]. Mutation in *Poc1a* (encoding protein of the centriole 1a) caused centrosome dysfunction which led to disorganized epiphyseal growth plates of their long bones [reviewed in (107)]. Seen from an evolutionary perspective, the youngest and most active retrotransposons are Alu SINEs comprising 11% of the human genome and unique to primates. Human endogenous retroviruses (HERVs) are a group of repetitive sequences that comprises 8% of the human genome (103). Regarding MSK physiology and pathology, HERV-W expression has been shown to increase in synovial fluids of human patients with osteoarthritis (108) and to play a role in human osteoclast fusion (109, 110).

Mobile genetic elements also shape the regulatory landscape of the human genome; for example, HERVs alone provide 320,000 transcription binding sites, Alu elements provide numerous splicing donor sites, whereas LINE-1s contribute to the generation of retrogenes and probably cause somatic mosaicism. Under normal conditions the majority of repetitive sequences are methylated, thus not transcribed. Under stress conditions and in pathological states (e.g. viral infections, inflammation, cancers) the expression status of mobile elements is altered and repetitive sequences are transcribed which can change the transcriptional regulation of genes and could lead to genetic instability (111). Even though mobile genetic elements form half of the human genome, their role in transcriptional regulation in skeletal diseases awaits further elucidation.

## Regulatory Interactions Between Enhancers and Their Target Genes

SNPs and other variants in non-coding regions can potentially influence the binding affinity of transcription factors and consequently change the regulation of bone homeostasis. Recently, several comprehensive functional studies of SNPs in intergenic regions have combined bioinformatics data analysis followed by functional validation *in vitro* and in animal models (112–115). A targeted search for novel potentially functional SNPs in enhancers that associate with bone metabolism was performed in five independent cohorts including 5,905 patients (116). In this study, correlation of SNPs with gene expression and biological processes resulted in 15 novel SNPs in enhancer regions (116). Analysis of transcriptional binding sites in the vicinity of osteoporosis associated SNPs revealed that they could affect the binding affinity of common transcription factors (NFATC2, MEF2C, SOX9, RUNX2, ESR2, FOXA1 and STAT3) which may be affected by SNPs and are involved in bone metabolism (117). High-throughput assays to speed the identification of functional variants have been developed in the last decade. Massive parallel reporter assays allow for the testing thousands of candidate regulatory sequences through cloning to a reporter gene followed by deep sequencing and analysis of transcription activation. This technique has recently been used to test 1605 SNPs residing in haplotypes implicated in osteoarthritis (118), highlighting its value to accelerate SNPs functional tests and genetic prioritization.

SNPs in the enhancer regions can change transcription factor binding sites (TFBS) and thus influence transcriptional regulation in bone metabolism. A study identified 5081 osteoporosis-related SNPs residing in enhancers (119). Transcription factor enrichment analyses identified *EZH2* TFBS as a common binding site typical for osteoporosis associated enhancer SNPs (119). Comprehensive analysis combining integrative functional genomics and experimental validation methods was reported for functional assessment of osteoporosis risk locus on 1p36 (112). First, the authors prioritized a particular SNP (rs6426749) with functional genomics. They then confirmed with dual luciferase assays and CRISPR/Cas9 silencing that this SNP acts as an allele-specific enhancer regulating the expression of a lncRNA (LINC00339). The downregulation of LINC00339 increases the expression of an important regulator of skeletal development, CDC42 (112).

Moreover, Zhu et al. have performed a comprehensive analysis to explain associations between SNPs in a potential RANKL enhancer region located 100 kb upstream of the *RANKL* gene and risk for osteoporosis (113). They employed eQTL, high-throughput chromosome conformation capture (Hi-C), epigenetic annotation, and functional assays to show that several SNPs residing in non-coding regions exclusively correlated with *RANKL *expression. This study revealed that *RANKL* transcriptional regulation is mediated by a long-range super-enhancer.

The importance of long-range enhancers was also demonstrated for *SOST*. As already mentioned, in van Buchem disease, patients carry a homozygous 52-kb noncoding deletion that is essential for the transcriptional activation of *SOST* in the bone (27). Deletion of specific long-range regulatory element *Ecr5* in mice caused the elevated bone formation and higher bone mass implying that the *ECR5* long distant region is responsible for transcriptional activation of *Sost* in the adult skeleton (114). In an integrative study, Carey et al. searched for BMD associated SNPs that are enriched in lineage-specific pathways during osteoclast differentiation (115). An overlay between BMD GWASs and active enhancers in the myeloid compartment revealed that the PU.1 transcription factor network is important for osteoclast differentiation.

The above exemplifies that (a) identification of enhancers is important, and (b) the combination of GWASs with experiments on model organisms helps to decipher pathways for skeletal conditions.

## Overview of -Omics Technologies for Skeletal Diseases: Transcriptomics, Epigenomics, Proteomics and Metabolomics

Over the past few decades tremendous advances in -omics technologies (transcriptomics, epigenomics, proteomics and metabolomics) have greatly expanded our knowledge into the cellular and molecular diversity, and pathological mechanisms underlying many diseases, including those affecting mostly the skeleton like osteoporosis and other skeletal conditions (120). While each -omic technology possesses the potential to capture a snapshot in a cell’s lifetime or disease state, individually they lack the power and capacity to capture holistic and spatiotemporal changes that occur at both the cell and tissue level during disease pathogenesis and progression. Therefore, there is growing momentum towards concerted multi-omic studies in the effort to integrate and unify data from different -omics platforms and thus better encapsulate all of the multilevel molecular and functional pathways that underpin a particular disease. However, the unification of -omics data presents challenges in combining and interpreting multilevel data sets, which are inherently large, complex, and call for significant computational grunt coupled with high-end bioinformatics. There are advantages of single-omics based approaches described herein, each that have contributed significantly to our current understanding of bone cell function and skeletal disease.

### Transcriptomics

Until recently, transcriptomic studies (i.e. the global survey of RNA transcripts; usually mRNA) in bone and its cellular residents have traditionally relied on microarray-based platforms, such as Affymetrix and Illumina chips. These have queried average transcriptome levels of osteoblasts (121), osteoclasts (122) and osteocytes (123). Despite their abundance in the bone, osteocytes remain comparatively underrepresented due to technical complexities when accessing these deeply entrenched bone mechanosensors.

Next-generation sequencing techniques strongly impacted transcriptomics with the development of RNA-seq (124), which has progressively replaced microarrays enabling a deeper dissection of the transcriptomes of bone cells (125). RNA-seq has been pivotal to the development of a high-resolution transcriptome of the osteoblast, as well as to the better characterization of changes along osteoblast differentiation (112, 126–133). Furthermore, it has unravelled the transcriptomic changes occurring in human MSCs that may contribute to the age-related impairment in osteoblast formation and/or function (134), and to the development and progression of osteoporosis (133, 135–137).

One of the demurs in bulk bone transcriptome profiling are both the temporal and spatial nature of transcriptomic studies. Whilst the former can be controlled for with experimental design, the latter provides a bigger challenge when trying to disentangle the bone tissue specific signals from those stemming from others i.e., bone marrow *vs* blood. Studies utilizing same methodologies, but integrating gene expression data obtained from blood-derived cells showed vastly different results, highlighting the need for tissue specificity (68). Recently, Youlten et al. integrated a strategy wherein matched intra-sample controls were used to distinguish genes enriched for osteocyte expression compared to other tissues (138). Whilst this approach controls for possible tissue contamination, it may be monetarily prohibitive since it warrants repeat analyses of the samples. As such, correction for tissue heterogeneity is most often utilized (139). Future developments in combining analytical approaches promise attenuation of such limitations of bulk sequencing by deconvolution of separate tissue contributions.

In parallel, the coupling of RNA-seq with cell sorting methodologies has now provided an unprecedented opportunity to gain detailed insights into the transcriptome of bone resident cells at a single-cell resolution (125, 140). To date, only a limited number of studies have applied single-cell RNA-seq (scRNA-seq) to bone cells, to investigate the transcriptome of osteoblasts at a single cell level (126, 141–143). Currently, a limited number of RNA-seq studies have been applied to osteoclasts. Still, this technique has been instrumental in clarifying the cellular origin of osteoclasts, both in human and mouse (142, 144); a more detailed osteoclast transcriptome is now available (145). RNA-seq has also been employed to study the osteocyte’s transcriptome to understand how it is modulated by fluid flow mechanotransduction (146) or PTH signaling (147). Single-cell resolution uniquely enables the identification of rare cell types, such as, reversal cells (43, 46), osteomorphs (69) and osteomacs (148), and to accurately define cellular heterogeneity between cell populations.

Meanwhile, the development of third-generation sequencing technologies, such as single-molecule real-time sequencing, is nowadays already enabling an even more accurate characterization of cellular transcriptomes at a single-molecule level (149). Third-generation sequencing methods will also improve our currently limited understanding of how a myriad of molecular mechanisms globally regulate transcriptomics in bone biology.

### Epigenomics

There is rapidly growing appreciation of the intimate interplay that exists between genes, the environment, as well as the fine regulatory control afforded by genome-wide epigenetic modifications including DNA methylation and histone modification. These have considerable effects on the differentiation and functional activities of bone cells, and may thus underscore mechanisms of skeletal disease pathogenesis. Comprehensive GWAS models only explain a fraction of the observed BMD variation. This prompts researchers to consider both the genes of interest and their regulation. The most direct approach would be to measure the protein levels since it is those that are responsible for downstream effects. However, due to inaccessibility of bone tissue it can be challenging to determine protein levels in an *in vivo* setting especially when dealing with humans – and even more so for diagnostic purposes. As a surrogate marker for gene and protein expression, epigenomics, at least in theory, is a more accessible approach. When aiming for clinical applications, DNA methylation is of particular interest because: 1) it acts as a master-regulator of histone modifications that govern gene expression, 2) it can shut down or open up gene expression, 3) it reflects inheritance, lifestyle, and environmental influences, and 4) it is stable even during sample handling e.g. blood sampling (150–152).

Indeed, recently it was shown that DNA methylation analyses based on blood were able to, at least partly, match the DNA methylation profile found in bone specimens obtained from osteoporotic women as well as with BMD (153). Yet, despite its obvious advantages it has not, until now, found clinical use in the osteoporosis field. Unexpectedly, despite far larger cohorts, two other studies were not able to show strong links between blood DNA methylation profiles and BMD (154, 155). Yet, recent findings showed that DNA methylation levels of the *DCSTAMP* gene were reduced with age resulting in higher expression levels, and that this would stimulate human osteoclast formation and activity both in *in vivo* and *in vitro* (156, 157). Still, it is important to remember that investigations on DNA methylation profiles to predict osteoporosis are just beginning. Up until recently, cost effective tools to do such analyses were missing. In recent years, this has changed primarily due to the dramatic drop in costs for WGS as well as the development of array-based techniques covering more than 850,000 CpG sites. This development makes epigenome-wide association studies (EWAS) possible. Of note, the only EWAS study of BMD performed to date, based on whole blood samples, revealed negative findings (155), suggesting limitations driven by tissue specificity and/or limited power to identify epigenomic effects.

### Proteomics

Proteomics enable unbiased identification and quantification of the total protein inventory of a particular cell type or tissue. Compared with data arising from genomic and transcriptomic studies, proteomics is closer to the phenotype, and is therefore considered a more suitable and reliable approach for mechanistic studies, disease typing, and as biomarkers (158, 159). Mass spectrometric analysis of proteins from organisms with sequenced genomes is advantageous as it allows for their routine identification, and modifications in analyzed proteins to be detected simultaneously. Thus, mass spectrometry (160), in particular tandem mass spectrometry has emerged as a powerful technique for the parallel quantitation and identification of proteins, with quantitation broadly assigned into two categories: label and label-free proteomics. While mass spectrometry is not inherently quantitative, several labeling methods are now available that afford robust quantification (161, 162). The development of sophisticated ‘delayed normalization’ techniques such as the MaxLFQ algorithm in MaxQuant has enabled accurate proteome-wide label-free quantitation. However, label-free techniques remain less robust than labeled methods (163). To date, there have been a number of seminal proteomic contributions (both quantitative and qualitative) at whole bone tissue and cellular levels, especially in the context of osteoporosis. At the cellular level, proteomic studies of osteoblasts [e.g (164); reviewed extensively elsewhere (165, 166)] and osteoclasts are available [reviewed in (167)], but osteocytes remaining comparatively unexplored.

With respect to osteoclasts, most proteomic analyses were performed in the context of RANKL-induced osteoclast differentiation (168, 169). However, also the proteome of secreted proteins (i.e. the secretome) (169), lysosomal hydrolases (168), and those enriched on membranes and lipid rafts (170, 171) were analyzed. Unfortunately, only few identified proteins have been validated experimentally. Of these, the Na^+^/K^+^ ion transporter (Nhedc2), was confirmed to play a role in bone resorption *in vitro* (170). Quantitative proteomic studies arising from the Hoflack laboratory unveiled several additional modulators of osteoclast polarization and function, such as the Src tyrosine kinases (172), and actin and membrane bridging proteins such as the Cdc42 guanine nucleotide exchange factor FGD6 (173) and ARAP1 (ArfGAP with RhoGAP domain, ankyrin repeat and PH domain-containing protein 1), the latter confirmed in mouse (174). Thus, quantitative proteomic approaches offer opportunities to uncover new molecules whose functions previously remained unassigned to bone. These may represent new therapeutic targets for the management of skeletal diseases.

### Metabolomics

Whereas the above mentioned -omics platforms are now mainstay in systems approaches to the study of skeletal diseases, the application of metabolomics (i.e. the study of small molecule chemicals, such as lipids, amino acids, short peptides, nucleic acids, sugars, alcohols, or organic acids) remains in relative infancy. The importance and utility of metabolomics in the bone field has, however, gained increasing appreciation in recent years, particularly towards its largely untapped potential to identify novel biomarkers of bone turnover/metabolism in skeletal disease settings such as osteoporosis [summarized in Yang et al. (120)]. As with other -omics technologies, metabolomics utilizes advanced analytical chemistry and statistical methods combined with bioinformatics to analyze the total metabolites within a cell, tissue, biofluid, or organism (175). Metabolites can be classified as either (i) “primary metabolites”: i.e. synthesized endogenously, or (ii) “acquired metabolites”: i.e. from dietary intake such as essential amino acids (phenylalanine, histidine, isoleucine, lysine, leucine, methionine, threonine, valine, and tryptophan) and vitamins (vitamins A, B, C, D, E, and K).

The inherent complexity in detecting and measuring different classes of chemicals that constitute the metabolome, in scales of magnitude larger than both the genome and proteome, necessitates wider and more sophisticated equipment. Such may be nuclear magnetic resonance spectrometers, mass spectrometers, gas chromatography, and liquid chromatography that are often employed in combination (See (175) for an extensive review). There are also different approaches to metabolomics experiments depending on the underlying questions being asked with the most common being targeted and untargeted approaches. While targeted metabolomics is widely applied in clinical applications for biomarker detection, untargeted metabolomics enables an unbiased approach to survey thousands of metabolites and has been the method of choice to compare the metabolomes of both humans (176–183) and rodents (184) in the context of osteoporosis. Although the number of metabolites tested to date (<2000) represents only a fraction of those circulating in plasma, serum, urine, and other biofluids, measurable differences in several amino acid and lipid metabolites have been detected, including increased glutamine (179, 182) and decreased proline (181) in menopausal women with low BMD. Whilst vastly underrepresented compared to other -omics technologies, as we move further towards an integrative multi-omics and holistic approach to skeletal diseases, the number of metabolomic studies is primed to accelerate and will undoubtedly uncover hitherto unappreciated but important metabolites that contribute to the regulation of skeletal homeostasis and disease.

## Overview of –Omics Data Resources From Human Bone Tissue

Since bone is a relatively inaccessible tissue, few -omics data resources are available; more specifically, data related to chromatin/DNA structure are missing. A summary of -omics data resources originating from human bone tissue is presented in **Table 2**.

**Table 2:** Overview of –omics data resources from human bone tissue by technology.

Description (author)	Number of samples	Availability of data	assessment type: global or targeted
Proteomics			
Immunological quantification of targeted proteins from postmenopausal iliac bone biopsies (185, 186)	56	Authors/publication	Targeted (SOST, DKK1, sFRP3, WIF1)
Western analysis of postmenopausal intertrochanteric bone biopsies (25 osteoporotic with fracture + 29 with OA) (187)	54	Authors/publication	Targeted (DKK1, β-catenin)
LC-MS analysis of young adult alveolar bone from two healthy females and two males (aged 15−21 years) (188)	4	PRIDE Project PXD011524	Global
Stable isotope labeling by amino acids in cell culture (SILAC) analysis of primary cultured human osteoblasts co-cultured with human umbilical vein endothelial cells (HUVECs) (189)	2	PRIDE Project PXD011844	Global
Shotgun proteomics (LC-MS) of archeological human bone from 4 adults and 2 infants (190)	6	PRIDE Project PXD006256	Global
LC-MS/MS analysis of cranial suture samples stripped of periosteum from 5 infants (ages 3–12 months) (191)	10	PRIDE Project PXD003215	Global
LC-MS/MS analysis of alveolar bone and dental cementum from 5 females and 2 males ranging from 20 to 30 years old. (192)	7	PRIDE Project PXD000420	Global
Transcriptomics			
RNA-seq of transiliac bone biopsies and subchondral femoral head samples (Prijatelj et al.) publication in progress	121	Authors	Global
eQTL analysis of transiliac bone biopsies (Prijatelj, Reppe et al.) publication in progress	76	Authors	Global
RNA-seq of purified osteoblasts from male iliac bone biopsies (127)	6	Authors/publication	Global
Microchip RNA profiling of postmenopausal transiliac bone biopsies (193)	84	EMBL-EBI repository, ID: E-MEXP-1618.	Global
PCR based and microchip profiling of postmenopausal iliac or femoral bone biopsy ncRNAs. (194)	84 + 18	Authors/publication	Global
Microchip RNA profiling of 19 spine and 5 iliac crest bone biopsies from 13 male donors. (195)	24	EMBL-EBI repository, ID: E-MEXP-2219	Global
Microchip profiling of postmenopausal intertrochanteric bone biopsies (10 with OA + 10 osteoporotic + 10 autopsies from controls) (196, 197)	30	Authors/publication	Global
PCR profiling of postmenopausal intertrochanteric bone biopsies (25 osteoporotic with fracture + 29 with OA) (187)	54	Authors/publication	Targeted, including mRNAs and miRNAs
PCR profiling of postmenopausal/male intertrochanteric femoral bone biopsies (49 with OA + 50 osteoporotic + 14 autopsies from controls) (198–200)	113	Authors/publication	Targeted
PCR profiling of femoral head bone biopsies from non-osteoporotic women (10 postmenopausal + 7 pre-menopausal) (201, 202)	16	Authors/publication	Targeted (>150 genes)
PCR profiling of male iliac crest bone biopsies (9 osteoporotic + 9 healthy) (203)	18	Authors/publication	Targeted
PCR profiling of elderly male femoral head bone biopsies (12 with osteoporosis/fracture + 10 with OA) (204)	22	Authors/publication	Targeted
PCR profiling after fracture of male and female femoral bone biopsies (45 with fracture/osteoporosis + 15 with fracture/non-osteoporotic) (205)	60	Authors/publication	Targeted
miRNA profiling of postmenopausal femoral neck bone biopsies (6 with osteoporosis + 10 with OA) and primary cultured osteoblasts from knee (n=4) (206)	16	Authors/publication	Global
miRNA profiling of postmenopausal femoral head biopsies (27 with fracture + 27 with OA) (207)	54	Authors/publication	Global
mRNA and miRNA PCR profiling of postmenopausal or male femoral bone biopsies after fracture (20 osteoporotic + 20 non-osteoporotic) (208)	40	Authors/publication	Targeted
PCR profiling of postmenopausal and male femoral bone (6 osteoporotic + 20 with OA) (209)	12	Authors/publication	Targeted (172 genes)
RNA-seq of centrifuged postmenopausal iliac crest bone biopsies from denosumab treated or untreated donors (210)	30	GEO Accession: GSM4209348	Global
RNA-seq of explant osteoblast cultures from human patients with non-syndromic craniosynostosis (n=23) and controls (n=8) (211)	31	GEO Accession: GSM1333404	Global
Microchip profiling of transiliac crest bone biopsies from 9 patients with endogenous Cushings syndrome before and after treatment (212)	18	GEO Accession: GSE30159	Global
Microchip profiling of transiliac crest bone biospies from 7 patients with primary hyperparathyroidism before and one year after parathyroidectomy (213, 214)	14	EMBL-EBI: E-MEXP-847	Global
Microchip profiling of transiliac bone biopsies from 2 male controls and 2 male patients with clinically characterized Fibrogenesis imperfecta ossium (215)	4	GEO Accession: GSE43861	Global
RNA-seq of centrifuged postmenopausal iliac crest bone biopsies from young women (n=19), postmenopausal women treated with estrogen (n=20) and postmenopausal controls (n=19). (216)	58	GEO Accession: GSE72815	Global
Microchip profiling of osteoclasts treated with bisphosphonates (n=6) and controls (n=3) (217)	9	GEO Accession: GSM1537946	Global
Microchip profiling of primary osteoclast precursors differentiated with CSF-1 and RANKL or CSF-1 alone (115)	4	GEO Accession: GSE107297	Global
Microchip profiling of OA (n = 20) and non-OA (n = 5) knee lateral tibial and medial tibial plateaus subchondral bone biopsies. (218)	50	EMBL-EBI: GSE51588	Global
DNA methylation			
Microchip DNA methylation profiling of postmenopausal transiliac bone biopsies (219, 220)	84	Authors/publication	Global
Microchip DNA methylation profiling of postmenopausal femoral bone biopsies (221)	30	Authors/publication	Global
PCR/pyrosequencing of femoral head bone DNA from postmenopausal women/elderly men subjected to hip replacement due to fracture or OA and RNA expression analysis of RANKL, OPG and BGLAP (222)	21	Authors/publication	Targeted
Sequencing of bisulfite-converted femoral bone DNA from 32 males or females with fracture, of whom 16 were non-osteoporotic and RNA expression analysis of RANKL, OPG, SOST (223, 224)	32	Authors/publication	Targeted
Sequencing of bisulfite-converted femoral bone DNA from 20 postmenopausal women with fracture, of which 8 were non-osteoporotic, and RNA expression analysis of SP7, RUNX2, SOST, ERα (225)	20	Authors/publication	Targeted
Microchip DNA methylation profiling of mesenchymal stem cells from postmenopausal femoral head bone biopsies (22 with fracture and 17 with OA) and RNA-seq of MSC samples from 10 women with fracture and 10 women with OA (136)	39	Authors/publication	Global
RRBS of primary cultured osteoblasts (226)	2	GSM683881, GSM683928	Global
Microchip DNA methylation profiling, changes during monocytes to osteoclast differentiation (227)	6	EMBL-EBI: GSE46648	Global
Microchip DNA methylation profiling of femoral head trabecular bone biopsies from females (n=46) and males (n=2). (228)	48	EMBL-EBI: GSE64490	Global
Pyrosequencing of DNA from human osteoclasts generated from women ages 40 to 66 years. Differentiation, fusion, bone resorption, and *in vivo* characteristics were evaluated in the context of DNA methylation of *DCSTAMP* and *CTSK* (156, 157)	49	Authors/publication	Targeted
**Chromatin structure**			
Hi-C and RNA-sec of primary cultured human osteocytes (Hsu, Kiel et al.; publication in progress)	1	Authors	Global
Dnase1-seq, ChIP-seq (H3K4me3), 5C and RNA-seq of primary cultured osteoblasts. (229)	1	GEO Accessions: DNase1-seq: GSE29692, GSE32970; ChIP-seq: GSE35583; RNA-seq: GSE19090, GSE15805, GSE17778; 5C: wgEncodeEH002102	Global
ATAC-seq, RNA-seq and 3C analysis of osteoblasts and adipocytes derived from human bone-marrow MSC (230)	4	European Bioinformatics Institute (EMBL-EBI) Capture C: E-MTAB-6862; ATAC-Seq: E-MTAB-6834; RNA-Seq: E-MTAB-6835	Global
ChIP-seq and RNA-seq experiments in MSC and immortalized osteoblastic cells (hFOB 1.19) before and after differentiation. ChIP-seq was done for H2A.Z, H2Bub1, H3.3, RNAPII and CHD1 in differentiated FOB with control or CHD1 siRNA treatment (231)	35	GEO Accession: GSE89179	Global
DNase1-seq and microchip RNA profiling of immortalized osteoblastic cells (hFOB 1.19) before and after differentiation (232)	10	GEO Accession: GSE75232	Global
Chip-seq of primary cultured osteoblasts in which DNA was precipitated with 11 different histone antibodies (233)	11	ENCSR786NTC	Global
DNase1-seq of bones from female and male embryos (98 and 81 days, respectively) (234, 235)	5	ENCSR805XIF, ENCSR976XOY, ENCSR431UEM, ENCSR274SDO, ENCSR449HOQ	Global

The data was generated based on searches in PubMed and the following portals: ProteomeXchange Data http://proteomecentral.proteomexchange.org/cgi/GetDataset; Sequence Read Archive (SRA) https://www.ncbi.nlm.nih.gov/sra; Gene Expression Omnibus (GEO) https://www.ncbi.nlm.nih.gov/geo/; OmicsDI https://www.omicsdi.org/; SkeletalVis http://phenome.manchester.ac.uk/; Array Express https://www.ebi.ac.uk/arrayexpress/; ENCODE https://www.encodeproject.org/search/?searchTerm=bone. Search finished by May 6_th_ 2020.

To best exploit the power of the various -omics data, genetic alterations must be combined in order to understand the interaction between features like SNPs, chromatin structure, DNA methylation, coding transcripts, non-coding transcripts, metabolomics, and bone status/bone metabolism, ideally supplemented with functional studies in cells and model organisms. GWAS follow-up studies are necessary to interpret GWAS results and to infer the exact disease-causal variants, the genes they regulate, and the cells in which they act (9).

With Hi-C, the chromatin loops and topologically associating domains (TADs) can be mapped (236). The hierarchical organization of chromatin can be further detailed with Assay of Transposase Accessible Chromatin sequencing (ATAC-seq), which maps nucleosome-free DNA available for transcription (237, 238). Furthermore, DNA methylation, as well as genome variants, can influence binding of gene regulatory proteins, thereby regulating gene expression levels. The DNA methylation pattern is also associated with the three-dimensional structure of DNA (239). DNA accessibility peaks indicate regions available for transcription factor (TF) binding to histone modifications (e.g. H3K4me1, H3K4me3, H3K27ac, and H3K27me3) (240). In particular, H3K4me3 peaks highlight gene promoters while H3K27ac peaks mark active enhancer and promoter regions (241). Thus, to promote the understanding of the underlying molecular mechanism of bone metabolism, several -omics analyses should be performed on the same sample in cells from patients with osteoporosis and controls. Unfortunately, such comprehensive studies are still missing, and at best, analyses of two or three different -omics layers have been combined in the same study. Current searches are largely limited by the availability of comprehensive reference functional data sets and the emerging set of analytical tools for multi-omic analysis.

The various studies often have different designs and purposes, and therefore are not directly comparable. Sclerostin is a central inhibitor of the Wnt signaling pathway, and various parts of the Wnt signaling system have been associated with bone status in most types of -omics analyses including GWAS, e.g., β-Catenin and DKK1 in proteomics (187); SOST, DKK1, WIF1, CTNNB1, and WNT5B in transcriptomics (193, 196); FZD10, TBL1X, CSNK1E, WNT8A, CSNK1A1L, SFRP4, and SOST in DNA methylation studies (221, 223). Also, TGF-β signaling genes (187, 196, 198) and regulators of osteoclast function (187, 197, 199, 242) have been identified in more than one type of -omics analysis. Furthermore, a study of DNaseI hypersensitive sites during osteoblast differentiation identified changes in chromatin and expression of several genes within the Wnt and TGF-β signaling pathways (232).

Some GWAS studies have included eQTL results, but often transcript levels of genes near the resulting variants are neither associated with the allele frequency nor BMD (243). A recent study indicates that Hi-C type methods are well suited to identify the effectors of causal genomic variants (230). In this study, the chromatin capture technique was combined with ATAC-seq to map 46 BMD GWAS loci to 81 gene promoters in human MSC-derived osteoblasts. Consequently, several novel genes physically interacting at the three-dimensional (3D) genome level with the causal variants of BMD were identified.

From **Table 2** it follows that gene expression is available for a wider set of cells and tissues than other types of -omics data (9). However, to get a comprehensive understanding of the genomic changes underlying bone diseases, it is necessary to identify eQTLs with different effect sizes at different stages of cell differentiation (dynamic eQTLs). This applies to cells of MSC progeny as well as those of monocyte lineage.

## Availability of the Bone -Omics Data

To date, there is not one single online resource that has collected data from all the available -omics analyses done on human bone tissue. At best, the resources are scattered throughout several such outlets. Sequence Read Archive (13), wherein high-throughput sequencing data is curated, is one of those (244). Even though the database itself is rather large, advanced search functionality built into it allows for easier navigation through the contents, which can be used to discover human bone tissue derived data. SRA is complemented by Gene Expression Omnibus (GEO) that may also host these same datasets, yet often enough, unique data can be found there as well (245). Since GEO is integrated into the National Center for Biotechnology Information (NCBI), like SRA is, its built-in search functionality allows for a similar navigation of the contents. ProteomeXchange is a portal dedicated to protein expression datasets (246), listed as one of the primary information resources by the Human Proteome Organization. Although human bone is a rare tissue in proteomic studies, bone-derived proteomic datasets are available on ProteomeXchange, although not in the same abundance as transcriptomic studies in other comparable resources.

Perhaps the closest approximation of “one-size-fits-all” collection of -omics datasets may be the OmicsDI platform, which acts as an integrational portal for proteomics, genomics, metabolomics, and transcriptomics datasets (247). The built-in search function in the portal has certain limitations e.g. improper implementation of the “NOT” operator in order to filter out undesirable results. The platform does though include a feature RESTful API for a possibility of implementing the search functionality within another website, or automating and curating the search results *via* a scripting language of choice, which may overcome the aforementioned limitations.

SkeletalVis is a portal devoted to exploration, visualization, linking of- and meta-analyzing skeletal transcriptomic data (248). Publicly available data resources (such as SRA, GEO, and ProteomeXchange mentioned before) are mined, undergo a QC procedure, and re– analyzed. The strengths of the platform include inter-experiment comparison options using signed Jaccard index, an approach that is also species-agnostic, visualizing datasets’ (dis)similarity using the t-distributed stochastic neighboring embedding, as well as other possible downstream analyses, whilst presenting the results in a user-friendly web interface.

Another user-friendly tool is a correlation browser to identify highly correlated transcripts in trans-iliac bone biopsies from 84 postmenopausal women (193). The correlation browser enables targeted searches among >260 million transcript correlations. This tool (http://app.uio.no/med/klinmed/correlation-browser/iliac-v2.0/) enables e.g. identification of candidate targets of transcription factors. It has been expanded to include mature miRNAs, thus also enabling identification of candidate interacting mRNAs/miRNAs (unpublished).

The flourishing of analytic *in silico* tools and software is remarkable, and increases the speed at which data can be processed and analyzed. However, with this abundance of possibilities, caution is warranted, as no single tool is comprehensive and none is infallible. It is imperative to understand the principles behind bioinformatic tools and to sensibly choose the most suitable one(s) for the purposes of the end user’s project(s).

## Cell Culture Models and Resources Available in the GEMSTONE Network

**Figure 4:**
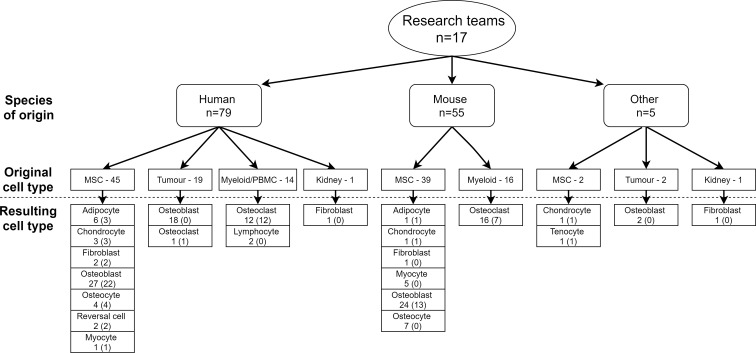
Collection of cellular resources available among 17 research teams of the GEMSTONE consortium.

Cellular models that accurately resemble/reflect the morphology and physiology of their originating/native tissue are pivotal tools to study bone biology and disease. However, the isolation of homogenous and functional primary bone cell populations remains technically challenging as most cellular residents are bound tightly to bone surfaces (i.e. osteoclasts and osteoblasts) or deeply entrenched in mineralized tissue (i.e. osteocytes), thus requiring specialized isolation methods. An amalgamated cellular repository: i) composed of a wide variety of primary and transformed skeletal cells; ii) derived from relevant skeletal tissue types, and iii) of multiple species of origin would therefore facilitate rapid and transparent inter-institutional exchange of cellular resources and methods. To this end, a survey of cellular resources within reach of this consortium (from 17 research teams) is illustrated in **Figure 4**. The scheme encapsulates both primary and immortalized cell lines, the majority being human or mouse origin (N=79 and N=55, respectively) but also includes rabbit, rat, and monkey. Multipotential MSCs derived from primary tissue sources are best represented (N=46 human) and (N=39 mouse) followed by tumor-derived osteoblast cell lines (N=18) and those of myeloid lineage/PBMCs (N=4 human, N=16 mouse). Osteoblasts are by far the most represented cell type (N=71) followed by osteoclasts derived from either human (N=13) or mouse origin (N=16), respectively. Not surprisingly, osteocytes are comparatively underrepresented (N=11), with only four derived from primary human sources. This cellular resource is not limited to bone cells, but also extends to neighboring and intersecting tissues/cell types.

Collectively, the shared cellular models will serve as a powerful resource towards accelerating the functional validation of new molecular targets potentially implicated with skeletal biology and disease. However, although bone cell cultures are an easy and useful tool for the discovery and/or functional validation of variants associated with bone cell differentiation, they do not fully reflect the *in vivo* situation. Complex interactions between cells and the surrounding matrix are often missing. Despite these drawbacks, cell cultures have several benefits including analysis on a specific cell type, excluding the influence of endocrine factors and complex tissue interactions, ease of gene manipulation, and they are cost effective. It can be seen from **Figure 4** that roughly half of the cellular resources used are immortalized/transformed cells. These are especially helpful because the cells in culture are rather uniform and can more readily be genetically modified, in contrast to many primary cell cultures. However, immortalized cells should be used with caution since the cell cycle is artificially altered through the transformation that may potentially affect cellular signaling. Where possible, confirmation in primary cell cultures could be of benefit.

We find that the most important step needed to make progress on the functional validation of GWAS findings using cell cultures, is that experts throughout Europe and the rest of the World share cells, protocols, and expertise with each other (249). Resource from **Figure 4** is a first step in this direction, but the GEMSTONE consortium will further substantiate this collaboration within the network, thus we strongly encourage other research groups to join us in this effort.

## Adding Complexity but Gaining Physiology: Use of Microfluidics and 3D Technology in Bone Research

To date, most of the data generated in laboratory settings are either 2D *in vitro* culture systems or animal models. Cell cultures often involve human cell types, whereby one or more cell lines are (co)cultured, which nonetheless lack some of the complexity as observed in real life. Hence, there is a pressing need for models that better reflect human bone metabolism, in which certain aspects can be included, such as a 3D environment, shear stress and chemotaxis. Approaches that have been taken to overcome these issues can be divided into 3 categories: 1) 3D printed bone scaffolds, 2) Bioprinting of scaffolds (containing cells), and 3) Microfluidic models (Organ-on-Chip).

3D printed bone scaffolds have been employed with numerous compositions, surface modifications, coatings, biomechanical properties, and porosities (250, 251). Initially, by implementing MSCs/osteoblasts to study osteogenesis, the complexity has gradually been increased by including e.g. endothelial cells/vasculature leading to vascularized bone tissue-engineered constructs (252). Taking this one step further, efforts in the leukaemia/bone tumor research field have led to models (partially) mimicking the human bone marrow microenvironment, enabling the study of complex pathology through simultaneous interactions between multiple cell types and their extracellular environment *in vitro* (253). More recently, 3D bioprinting tools have become available, which allow for generating a 3D structure with the cell type(s) of interest included in the printing process (254, 255). 3D scaffolds have been widely used as *in vivo* bone regeneration models, but their translation value for human bone biology is yet unclear.

Over the last decade, simple microfluidic set ups have evolved into multi compartment-based chips, often coined Organ-on-Chip (OoC), in which relevant physiological aspects can be studied, including shear stress and chemotaxis. Many OoC models have studied shear stress for endothelial cell function, but evidence is growing that also MSCs, osteoblasts and osteocytes perform better under fluid flow as evidenced by increased proliferation and altered marker genes expression (256–258). This suggests that OoC models may better reflect biology than conventional cultures. Similar to 3D printed scaffolds, OoC approaches have also led to employing more complex microenvironments, for example breast cancer-derived bone metastases or the so-called Bone Marrow-on-a-Chip (259, 260). The small format of OoCs may also allow for personalized medicine initiatives and for compound screening, as small amounts of cell numbers and materials suffice for cell culturing (261, 262).

Thus, despite the technical challenges ahead of us, 3D (bio)printing and microfluidics are at the forefront of a new era that may enable us to better recapitulate the physiology of bone tissue. The outcomes from various bone-related GWAS and clues from monogenic disease states has yielded a valuable list of target genes to scrutinize in a 3D environment with all the relevant physiological cues. With the use of primary cells or induced pluripotent stem (iPS) cells, the toolbox expands to generate a ‘bone-on-a-chip’ that relates to the disease or condition of interest. Ultimately, this may lead to improved therapeutic opportunities for bone metabolism and tissue engineering.

## Animal Models

### Laboratory Mouse as a Model Organism in Skeletal Diseases

*Necessity of animal models:* Functional validation of bone GWAS loci is performed frequently through genetic modifications in model organisms, with analysis of the resulting skeletal phenotypes. The bone- and joint-specific extreme phenotype screens in knockout mice [incl. the collaborative cross mouse panel (263)] identify novel pathways regulating normal bone and cartilage development, maintenance and resilience, thus uncovering new genetic determinants of disease, and provide *in vivo* models to investigate novel treatments. Skeletal development and maintenance are regulated by local and systemic factors; this complexity cannot be modelled *ex vivo. In vitro* techniques do not offer an alternative because skeletal development and bone turnover are dynamic processes, whilst mechanical forces, movement and tissue responses to injury modify bone maintenance. Mice are used extensively in studies of the skeleton. Key molecules that regulate cartilage (e.g. Wnt/beta catenin, Ihh, PTHrP, Sox9, FGFR3) and bone (e.g. Wnt/beta catenin, Runx2, FGFR1, osteocalcin, osterix, OPG, RANKL, TRAP, cathepsin K, TNF) in mice have the same functions in man, and human genetic disorders causing abnormalities of cartilage and bone are recapitulated in genetically modified mice. Similarly, endocrine and metabolic control of bone and cartilage is faithfully preserved in mice. This way, transgenic mice overexpressing human genes constitute valuable systems for the modeling of human diseases.

*Strategies for genetic manipulation:* Microinjection of the exogenous gene into the pronucleus of fertilized oocytes has been a standard method for the generation of transgenic mice, whereas a limitation of the technique is the random integration of the injected DNA into the genome. To achieve a physiologically relevant expression pattern, large genomic human transgenes of approximately 200kb usually provide copy-dependent expression levels, regardless of position effects (264), as also shown in humanized transgenic mouse models of osteoporosis expressing human RANKL (265).

On the other hand, the physiological role of a gene in mammalian homeostasis can be investigated in knockout mice with global gene deletion through homologous recombination in embryonic stem cells. In this way, rare genetic human skeletal diseases can be modeled in mice. If the knockout mice develop embryonic lethality, the conditional knockouts and inducible knockouts produced using the Cre/loxP recombination system allow gene loss in specific cells and tissues (spatial) and at the desired time (temporal). Transgenic mice expressing Cre recombinase under bone specific promoters offer excision of the target gene only in cells of the skeletal system (266). Furthermore, during the last years gene-editing technologies including zinc finger nucleases, TALENs, and CRISPR/Cas9 have offered the ability to generate specific alterations in the genome such as insertions, gene knockouts, and point variations (267). No matter how the mouse models were generated, it is important to consider their genetic background as this may be important for analyzing specific traits. In fact, pure genetic backgrounds (backcrossed for at least 10 generations) are preferred over mixed backgrounds to exclude effects that may stem from the genetics of the mouse instead of the targeted gene knockout. Apart from the reverse genetics approaches, forward genetics enable the identification of causal variants through analysis of mutants displaying bone phenotypes, allowing for the identification of genes critically involved in bone homeostasis (268).

*Analysis of genetically altered mice:* When analyzing the consequences of genetically manipulated animal models, it is worthwhile to consider which cell types are affected. Traditionally, the focus is on the classical members of the basic multicellular unit, namely the osteoclast, osteoblast, and osteocyte. However, recent findings from human bone tissue studies imply that other relevant cell types must also be considered, including bone lining cells, reversal cells, bone remodeling compartment canopy cells, and the bone marrow envelope as osteoblast progenitors (43, 44, 46). These cell types and structures have also been identified and characterized in mouse, rabbit, and sheep animal models (269–271). Moreover, scRNA-seq analyses now provide information into the subpopulations of osteoblasts and osteoclasts. Thus, when interpreting the bone phenotype of an osteoblast or osteoclast “specific” knockout, it is worth considering the possible influence of these cell types on the phenotype. With respect to osteoclasts, there may also be differences in their resorptive characteristics (272), they are affected by gender and age (156, 157, 273, 274), and there might be a variation in their source “residence” and precursors (274–277). We would therefore encourage scientists to consider these nuances when interpreting bone phenotypes resulting from genetic manipulation in animal models.

Bone phenotyping can be done at various depths. The ultimate test to determine bone strength, ideally at various skeletal sites, is by performing biomechanical testing. Additionally, micro-computed tomography (µCT) is a useful tool to determine cortical and trabecular bone microarchitecture. To obtain information about the presence and function of bone cells, dynamic bone histomorphometry or at a more advanced level, time-resolved 4D µCT, is useful. In this way, the number of cells can be quantified, and by applying fluorescent labels to the mice, even the bone formation rate within a given time can be estimated. Measuring bone turnover markers in the serum can additionally provide information about the activity of bone cells. Some researchers have developed standardized high-throughput screens for knockout mice at various depths, taking advantage of the International Mouse Phenotyping Consortium (278) seeking to screen phenotypes across KO models of all genes, and specifically for high-throughput screening of musculoskeletal phenotypes within the Origins of Bone and Cartilage Disease consortium (279).

*Standardization of bone phenotyping in mice:* Notwithstanding the experimental approach, it is of utmost importance to establish resource-sharing standards across research groups for the analysis of bone phenotypes in laboratory mice. Here we propose fundamental principles and outline a unifying methodology.

1. Use µCT to analyse bone morphometry in 3D in accordance with the established methodology and nomenclature (280).
2. Analyse cortical and trabecular bone separately, since growing evidence suggest that genetic variation may influence these compartments differently (243, 281).
3. Analyse at least two skeletal sites, one appendicular (e.g. femur) and one axial (e.g. lumbar vertebra), since different skeletal sites may be under different genetic regulation.
4. Perform *in vivo* scans to allow longitudinal studies in the same animal. In the past, this technique lacked in quality and resolution, but latest *in vivo* scanners are now offering an image quality comparable to that of *ex vivo* systems.
5. Perform biomechanical testing to directly assess bone strength. At the femur and tibia, 3- and 4-point bending tests are useful whereas compression tests are recommended for vertebral bone.
6. Perform dynamic histomorphometry, which is highly recommended to assess differences in bone turnover. Calcein double labeling and TRAcP staining are recommended to evaluate bone formation and resorption, respectively. To reach higher levels of standardization and reproducibility between groups, studies should be conducted in accordance with the standard methodology and nomenclature (282) and/or use the unified methodology presented by the Rowe’s group (283).
7. Use alizarin red/alcian blue staining to analyze the skeleton at the embryonic stage.

### Zebrafish as Animal Model for Functional Studies of Candidate Loci in Skeletal Diseases

**Figure 5:**
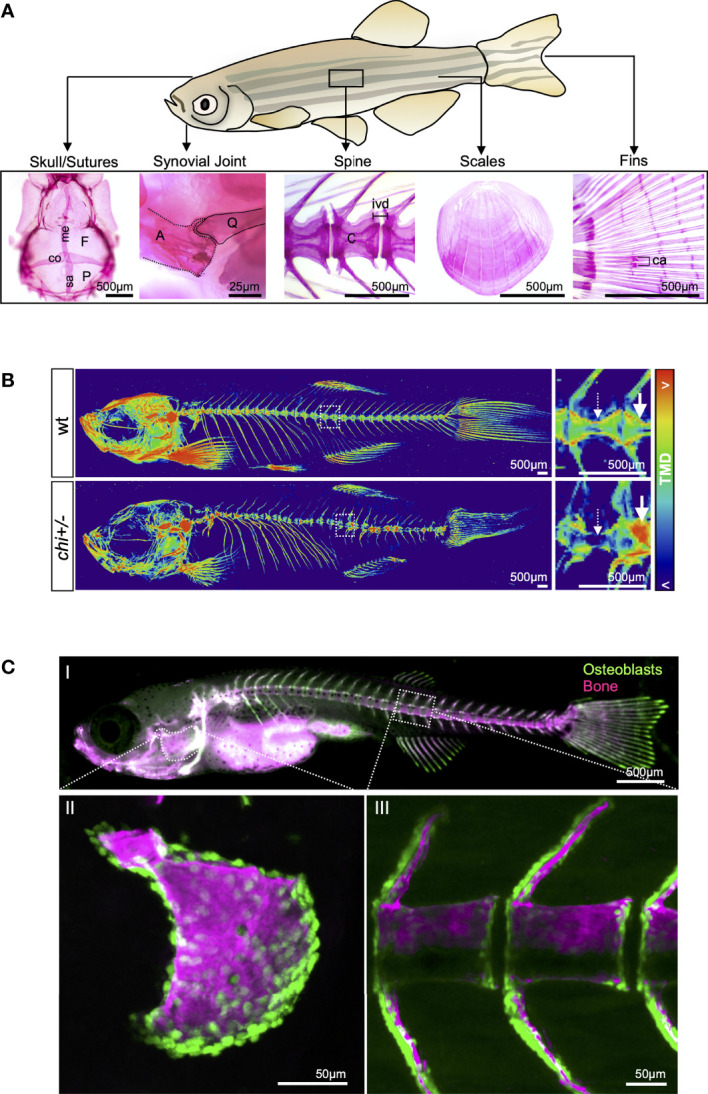
Zebrafish: a versatile animal model to study bone associated diseases. **(A)** Illustration of an adult zebrafish showing examples of bones through the zebrafish body used to model human diseases. Bones are shown stained with Alizarin Red S: skull (F = frontal or metopic; P = parietal), cranial sutures (me= metopic; co= coronal; sa= sagittal); synovial joint (A= anguloarticular; Q= quadrate); spine (C= centrum; ivd= intervertebral disc); scales and fins (ca= callus formed after fractures). Pictures were taken using a stereomicroscope (Leica MZ10F). **(B)** 3D volumetric renders from µCT images of wild-type (wt) and *chi+/-* (model for OI) adult skeleton, color-coded to show variations in TMD (red= higher TMD values; blue= lower TMD values). Regions within the dashed boxes are shown in higher magnification. Note the reduced and uneven TMD distribution in the bones of *chi-/-* (arrows and dashed arrows). Example of live imaging in juvenile zebrafish. **(CI)** Juvenile zebrafish carrying the transgene *Tg(Ola.Sp7:nlsGFP)zf132* (305), labeling osteoblasts in green, and live stained with Alizarin Red S, labeling mineralized bones in red (here shown in magenta). The picture was taken under a fluorescent stereomicroscope (Leica MZ10F). The operculum **(CII)** and part of the vertebral column **(CIII)** were live imaged under a confocal microscope (SP5 Leica) to show the structures in detail. Note single osteoblasts (green) contouring the mineralized bone (magenta) in II and III. Scale bars values are indicated in each picture.

The teleost *Danio rerio* (zebrafish) is a small size freshwater fish of relatively simple and cost-effective maintenance. It has emerged as an advantageous model organism for the study of vertebrate gene function, also allowing drug and genetic throughput screenings (**Tables 3**, **4** and **Figure 5**) (306, 307). Zebrafish have been of interest in bone research as their skeletal system shows high homology with human’s, exhibiting osteoblasts, osteocytes, osteoclasts, and chondrocytes (308). Embryos are translucent and develop fast, showing chondrocytes and osteoblasts at three days post-fertilization (dpf) (309–311) and osteoclasts at around 14 dpf (312, 313). Functional genetic studies of bone and cartilage are commonly performed in larval and juvenile zebrafish, where 3D skeletal analyses can be performed *in vivo* longitudinally using transgenic lines labeling specific bone-related cell types allowing cell trackability (314, 315) (**Figure 5**). The zebrafish vertebral column comprises the major skeletal component; it is fully formed by around two months post-fertilization (316, 317). Similar to mammals, it is formed by vertebral bodies separated by intervertebral discs; however, vertebrae show limited trabeculation and are mostly composed of dense, compact bone (318). Despite being an aquatic organism, loading patterns of the vertebral column are similar to bipeds, and can be experimentally controlled by varying applied forces through water resistance while swimming (319).

**Table 3:** Comparison between mouse and zebrafish.

Characteristic	*Mus musculus (mouse)*	*Danio rerio (zebrafish)*
***Maintenance and breeding***		
*Cost and time for animal husbandry*	***Modest*** (£2.5 per week, 8 animals per cage)	Low (£4 per week, 20 animals per tank)
*Facility housing and space required*	High	Low
*Sexual maturity*	~6-8 weeks	~6-9 weeks
*Life span*	~2 years	~3.5 years
*Fertilization and development*	Internal	External/fast development
*Control of fertilization time*	Limited	High, upon exposure to daylight
*Number of offspring per female*	Up to a dozen per month	Up to 200 per week
***Genomics***		
*Size of genome*	GRCm38.p6: ~3.49Gbp	GRCz11: ~1.67 Gbp
*Number of chromosomes*	2n=38+2(X/Y)	2n=50
*Coding genes*	24,278 (MGI, July 2020)	25,592 (GRCz11, May 2017)
*Non-coding genes*	16,074	6,599
*Coding genes with human orthologs*	~76%	~71%
***Genome engineering and transgenesis***		
*Genome manipulation*	Modest	Relatively easy
*Costs of establishing a stable line*	High	Low
*Forward genetic screening*	Yes (high costs)	Yes (modest costs)
*Reverse genetic screening*	Yes	Yes (modest costs)
*Mosaic (G0) screening*	Non-applicable	Yes (modest costs)
***Skeletal phenotyping and imaging***		
*in vivo imaging and cell tracking*	Available (modest)	Easy
First bones appear	E13.3 (chondrocytes); E15.5 (ossification centres)	3 days post-fertilization
*Imaging of early skeleton phenotype*	Modest (invasive)	Easy (translucent)
*Availability of exoskeleton?*	No (except teeth)	Yes (fins, scales)
*Visualisation of adult bone fracture*	Invasive	Non-invasive (fins) and invasive (vertebral column)
*X-ray (TMD)*	Easy	Easy (full body, relative TMD)
*µCT (TMD)*	Easy	Easy (bone structure)
*Biomechanical* test*s*	3-point-bending, vertebral compression, nanoindentation	nanoindentation, vertebral compression
***Selected repositories for bone phenotypic data***	International Mouse Phenotyping Consortium (IMPC) (www.mousephenotype.org)	There is not a specific database available
	INFRAFRONTIER (www.infrafrontier.eu)	
	Origins of Bone and Cartilage Disease (OBCD) (www.boneandcartilage.com)	
	Mouse Genome Informatics- The Jackson Laboratory (www.informatics.jax.org) https://bonebase.lab.uconn.edu/	

**Table 4:** Zebrafish genetic models for human skeletal diseases.

Human Disease/condition	Zebrafish genetic models	References
Osteoporosis	atp6V1H	(284)
	sp7/osterix	(285)
Osteogenesis imperfecta (OI)	col1a1a (chihuahua)	(286, 287)
	col1a2	(288)
	bmp1a (frilly fins)	(289)
	plod2	(290)
	sp7/osterix	(285)
	pls3	(291)
Craniosynostosis and ectopic sutures	cyp26b1 (dolphin and stocksteif)	(292)
	tcf12 and twist1	(293)
	fgfr3	(294)
	sp7/osterix	(285)
Fibrodysplasia Ossificans Progressiva	acvr1/alk2	(295)
Scoliosis	cc2d2a	(296)
	kif6	(297)
	c21orf59, ccdc40, cctc151, dyx1c1 and ptk7	(298, 299)
	col8a1a	(300)
Osteoarthritis	col11a2	(301)
	prg4	(302)
ectopic mineralisation (axial skeleton)	abcc6a	(303)
	enpp1/enptd5	(304)

*Analysis of genetically altered zebrafish:* Alizarin red (*in vivo* or *ex vivo*) and Alcian blue staining (*ex vivo*) are simple techniques that allow skeletal assessment from larval to adult stages (320) (**Figure 5**). Radiographs and µCT are commonly applied in adults, permitting longitudinal studies and post-mortem BMD (cortical bone density) calculations, respectively (286, 319). Higher-resolution µCTs (< 5µm voxel size), used to study osteocyte lacunar parameters (number, orientation and shape) (287, 294, 314, 319), are therefore suitable to investigate the effect of osteoporosis genes in the 3D organization of osteocytes. The superficial position of the skulls, fins, and scales also permit the acquisition of *in vivo* and longitudinal images using transgenic lines, making them attractive systems for drug screens (315), and studies of skeletal development, regeneration (fin amputation, scale plucking, and skull trephination) and bone fragility (fin and scale fractures) (321–325). Similar to other model systems, the assessment of bone quality is possible through 2D static and dynamic bone histomorphometry and vibrational spectroscopy methods (e.g. Fourier transform infrared spectroscopy and Raman spectroscopy) (287, 319). Bone material properties and fracture risk are retrieved through nano-indentation or through compression forces applied on entire segments of the vertebral column (287, 314, 319, 326).

*The zebrafish *vs* the human genome*: Over 70% of human genes have at least one zebrafish ortholog (327). Due to whole-genome duplication events during the zebrafish evolution, approximately 25% of human genes have more than one ortholog in zebrafish (328, 329). Despite this teleost-specific event’s adding more genes and complexity for functional genetic tests in zebrafish, it can be also seen as an advantage for the study of gene function, as genetic manipulation of individual paralogs might bypass lethality and enable assessment of larval to adult skeletal phenotypes. Furthermore, the aquatic environment contributes for long term survival of those fish almost completely lacking bone from genetic manipulation (288). Large forward genetic screens, using the chemical N-ethyl-N-nitrosourea (ENU) as a mutagenesis agent, have provided models for several skeletal diseases over the years (330–333). The zebrafish mutation project aiming to generate a knockout allele for each protein-coding gene made many mutants available to the scientific community (329). For example, the *chihuahua* (*chi*/+) mutant which recapitulates the skeletal phenotypes exhibited in human classical dominant OI (286, 287, 334) (**Figure 5**). Recently, avenues for new therapeutic discoveries using adult zebrafish have been demonstrated through treating the *chihuahua* with 4PBA and TUDCA chemical chaperones (334). Other zebrafish genetic models for MSK are exemplified in **Table 4**.

*Zebrafish genetic models:* Models to study osteoporosis have only been developed recently in zebrafish, concomitantly with the study of the ageing zebrafish spine (335, 336). Recently, Kague et al. provided strong support of osteoporosis in zebrafish, showing that aged zebrafish spines display increased susceptibility to fractures and bone quality deterioration (tendency towards reduction of BMD, increased bone mineral heterogeneity and poor collagen organization) (337). Genetic manipulations could provide consistent and compelling models for osteoporosis, as shown in **Table 4**. Functional evidence in osteoporosis through zebrafish studies is exemplified with *ATP6V1H* (284), *SP7* (285), and *LRP5* (338). Co-segregation between a mutation in the *ATP6V1H* gene and osteoporosis was reported in a human three-generation pedigree and functional studies performed in zebrafish using CRISPR/Cas9. *atp6v1h* zebrafish mutants showed a reduction in mature bone, reduced bone mass and density, providing functional evidence of *ATP6V1H* in osteoporosis (327). *SP7/OSTERIX* has been associated to OI (339), Paget’s bone disease (340) and osteoporosis by GWAS (341). Zebrafish *sp7* mutants showed reduced BMD, spontaneous fractures, abrogation of osteoblast differentiation, reduction of number but increase in osteocytes’ volume, and abnormal bone material properties (285, 337). Similarly, when *lrp5* was mutated in zebrafish it led to reduced BMD, bone volume and cortical thickness, reminiscent of osteoporosis (338). Despite the limited number of zebrafish models of osteoporosis available to date, recent studies (337, 338) have provided additional evidence that zebrafish are natural models for osteoporosis during ageing and zebrafish mutants for genes associated to BMD can recapitulate osteoporosis phenotype, therefore, placing zebrafish as a parallel model along with mice to functionally study and validate osteoporosis associated genes.

*Strategies for genetic manipulation*: Among genome editing technologies, the CRISPR/Cas9 system has become the most widely used in zebrafish as it provides a straightforward, efficient, and accurate gene editing (342–344). It has contributed to an exponential increase in the number of mutants involved in skeletal phenotypes published in the last years. However, the time required for the generation of homozygous mutant lines (up to one year) is a limiting factor when planning to test the vast number of genes harbored in GWAS identified loci. Proof-of-concept was achieved by Watson et al. showing that CRISPR genetic screening through G0s (mosaic fish) can be used for evaluation of larval to adult skeletal phenotype without the long waiting time to generate a stable homozygous mutant. The authors tested two genes (*plod2* and *bmp1a*) involved in OI, and by comparing them with homozygous mutants showed adult CRISPR G0s (crispants) to recapitulate homozygous inbred phenotype in the skeletal system (343). The same approach was used to compare *lrp5* crispants versus knockouts, showing similar results in both groups and validating the use of zebrafish crispants to study genes coupled to osteoporosis (338). Therefore, G0s (crispants) provide loss-of function genetic screening in zebrafish allowing to test in parallel all genes harbored in GWAS associated loci. While deep phenotypic and molecular characterization can be performed in mutant lines, crispants represent an efficient *in vivo* platform and one of the greatest advantages of using zebrafish to boost identification of variants with high changes of causality in osteoporosis.

**Figure 6:**
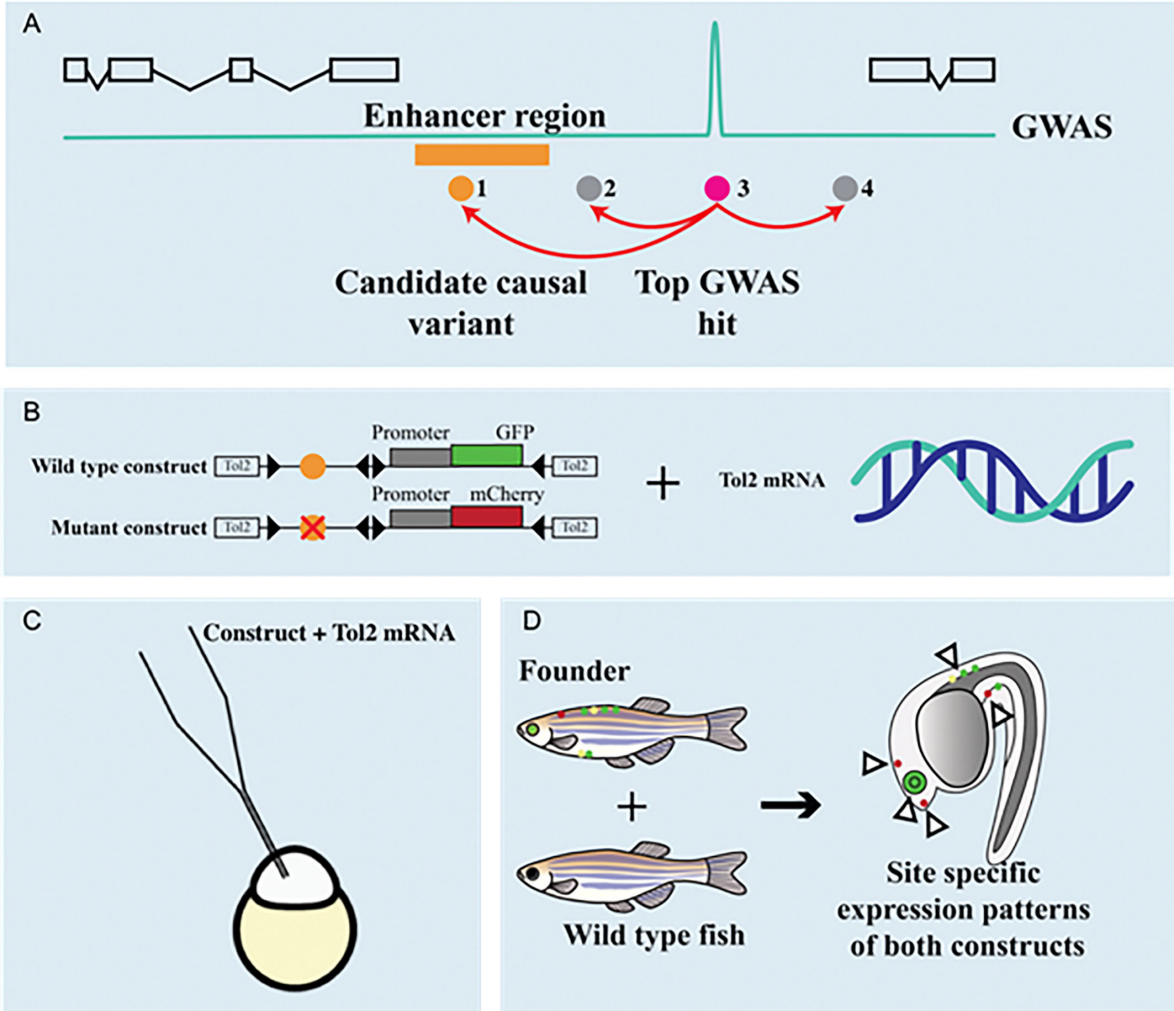
*In vivo* functional validation of non-coding variants using zebrafish. **(A)** An example of a top variant (3-magenta) identified through GWAS. The variant 3 is in linkage disequilibrium (red arrows) with other non-genotyped variants (1-orange, 2 and 4- grey). By combining information from other -omics, functional evidence is provided by showing an enhancer region overlapping the variant 1 (orange). An in vivo functional approach can be performed using zebrafish, were all organs and tissues are studied at the same time. **(B)** For this, each allele is cloned upstream of a generic promoter and a reporter (either GFP or mCherry) within a construct flanked by Tol2 transposable elements. Tol2 mRNA (transposase) can be easily synthesized. **(C)** Both or individual constructs in combination with the Tol2 mRNA are injected in the zebrafish embryo (one cell stage represented), leading to a DNA transposon-mediated integration in the zebrafish genome. **(D)** Results can be observed already in G0s (mosaic, founders), which when crossed to a wild-type zebrafish will contribute to germline transmission and generation of transgenic lines showing tissue-specific expression of the reporter (arrowheads). This system could be used to screen a high number of variants using G0s and precise quantification of reporter variability.

Besides targeting coding genes for functional studies, zebrafish are suitable to study lncRNAs and cis-regulatory regions. LncRNAs have been reported during embryonic development and in adult tissue, emphasizing their conserved biological function despite their limited sequence conservation (345–348). Transgenic analyses of cis-regulatory regions in G0s are possible through the Tol2 transposase system, whose high efficiency guarantees genomic integration and rapid delivery of putative sequences, allowing to test non-coding variants and their enhancer activity during zebrafish development (77), with evaluation of multiple tissues at once (i.e. bone and cartilage) (349). This system allows to test high numbers of conserved and non-conserved sequences (349–352), and could be improved towards the development of throughput systems to test enhancer activity *in vivo* (**Figure 6**). This strategy would benefit GWAS hits of difficult interpretation, within non-coding sequence, as demonstrated by identification of an enhancer element in the vicinity of *BMP2*, identified through GWAS as associated with non-syndromic craniosynostosis (353). Evolutionary conservation is frequently used as a filter to narrow down the number of sequences submitted to functional evaluation. While CRISPR/Cas9 systems can also be applied to cause large deletions (354) and to test conserved sequences; non-conserved sequences can still be tested for enhancer activity, due to degenerated binding motifs of transcription factors. It has been demonstrated that human sequences when inserted into the zebrafish genome lead to reliable enhancer activity in relevant tissues (349, 350).

Therefore, zebrafish provide a wide-ranging toolbox to functionally test coding and non-coding sequences identified in human studies which could be easily incorporated as a post-GWAS pipeline for osteoporosis.

## Establishing Data and Resource Sharing Platform

A cornerstone of science is the ability to replicate results. In the early 2010s the “replication crisis” was formulated to drive attention to the problem of inability to reproduce many findings (355). Part of this issue is also an increasing volume of scientific research being published (356). As part of the scientific method involves creating a conjecture, which relies on observations and prior knowledge, the mentioned crisis presents a problem that is two-fold: first, even though the information age makes it ever so simple to allocate desired material, the direct cause is also the exponential growth of the amount of data itself (357). Second, even when one is able to find wanted knowledge, the replication crisis should make researchers always second-guess the published results on their journey towards hypothesis creation. Thus, it would be prudent to build upon a service such as SkeletalVis and the “Musculoskeletal Knowledge Portal” [MSK-KP (358)], that allow not only -omics data resources to be available, but would also offer an overview, a “curation”, of results, whilst enriching them with other sources of relevant information. It is only through community effort that such advances are possible.

International Federation of MSK Research Societies (IFMRS) realized that there is a growing urgency for reproducible research using integrated -omics, similar to all disciplines in science. Along with the creators of relevant databases, data archives and knowledge-databases, IFMRS strived to identify common issues in database development, curation and management, to create data portals allowing reproducibility of singular -omics and integrated omics data (358).

IFMRS recently created MSK-KP, designed to facilitate functional studies of the genetic factors underpinning skeletal diseases, thus supporting the prioritization of genes and pathways by experimentalists. The ultimate vision of the MSK-KP was to consolidate -omics datasets from human and model organisms into a central repository that can be accessed by researchers to better understand the biological mechanisms underlying musculoskeletal disease and apply this knowledge to identify and develop new disease interventions. To realize this vision, the team from the Broad Institute (Cambridge, Massachusetts) was recruited, who had been instrumental in designing the Knowledge Portals for other complex diseases. Rather than simply serving as a repository for skeletal datasets generated by individual laboratories and large consortia, MSK-KP will provide a much-needed bridge between the statistical genetic, wet lab and clinical communities (358).

This effort requires making -omics data a resource on a web server that is publicly available to the scientific community, *via* an intuitive and flexible web interface that enables non-specialist users to mine and interpret -omics data easily (359). Thus, the summary of the results of existing and ongoing GWAS and PheWAS analyses is already deposited at the portal. At present, transcriptomics and epigenomics data are populated there. At the next stage, proteomics and metabolomics datasets will be added; in the future, bone-related lipidomics, microbiomics, spatial transcriptomics, and phenomics data will follow.

MSK-KP group will continue identifying, obtaining, curating and integrating various -omics datasets from the international MSK research community, and encourage data sharing through community collaborative spirit (358). Together with hosting the data from cellular experiments and animal models, as well as compound screens, the portal is supposed to integrate such data with bioinformatics resources. Part of the solution of appropriate integrating -omics datasets is a requirement for open sharing of scripts and codes for such analyses. Data provided to the MSK-KP should adhere to Findability, Accessibility, Interoperability, and Reusability (FAIR) principles (360). Besides archiving the data, IFMRS strives to provide guidelines on the integration of -omics datasets for the development of standardized analytical pipelines. This can move the skeletal genetics into the “post GWAS” era.

## Vision of the Future

This consensus statement aims to create a roadmap for using functional genomics to interrogate signals from human genetic studies for osteoporosis and other skeletal conditions. While the number of -omics-based studies in bone biology has exploded over the past decade, high throughput *in vitro* systems and rapid phenotyping of model organisms remain equally important in order to accelerate the functional validation of identified targets. Our effort nurtures such endeavors by expanding the wealth of knowledge and resources, represented by keen individuals and assets at their disposal, and promoting their exchange amongst participating institutions.

The unification of -omics data will create a wealth of new information towards the study of skeletal diseases (359). Besides increasing sample size, algorithms for standardization of sampling and sample processing, platforms for quantification, and data analyses should be agreed upon. Bone cell-type-specific resources might allow high-throughput massive parallel reporter assays that test the variants altering the activity of putative regulatory elements (361). Advances in computational analyses together with novel gene editing techniques that enable epigenetic manipulations at particular enhancer sites will further unveil relationships between non-coding SNPs and disease development.

Functional genomics studies of osteoporosis have limitations, mostly due to inaccessibility of bone tissue. In particular, not many resources are dedicated to osteocytes; since longitudinal studies of (especially human) specimens are limited, studies of bone loss rate are underrepresented. There are also limited ATAC-Seq and Hi-C datasets derived from bone cells, and it is still challenging to determine protein levels in bone in an *in vivo* setting, especially for diagnostic purposes. Furthermore, even though mobile genetic elements form half of the human genome, their role in transcriptional regulation in MSK diseases awaits further elucidation. While still vastly underrepresented compared to other -omics technologies, metabolomic studies of resident bone cells will undoubtedly uncover hitherto unappreciated but important metabolites. All this dictates our joint interest for the near future.

As we start to unravel the proteomes of bone cells and build towards more complete protein atlases, future approaches should be aimed at reducing sample complexity. Reducing ‘noise’ will thus enable precision mapping of proteomes at single organelle resolution, such as mitochondria or lysosomes. Similarly, rapidly evolving multiplex technologies such as imaging mass cytometry (e.g. Hyperion) and imaging mass spectrometry (e.g. Cell DIVE) coupled with spatial transcriptomic platforms will undoubtedly afford new and exciting opportunities to systematically map the molecular, cellular and spatial organization of musculoskeletal tissues at unprecedented resolution.

There is a need to continue close communication with the animal modeling groups to assure that *in vivo* studies cross-fertilize with cellular and translational ones, allowing the planning for functional validation of candidate variants to be taken concomitantly with genetic prioritization. Alongside the plethora of tools available for functional studies in cell culture and mice, zebrafish provide alternative and advantageous solutions for rapid functional screening of coding and non-coding sequences, and translation of genomic findings to therapeutics. The combination of functional expertise made accessible through our collaborative group, allows us to discuss data access, curation, and sharing in a collaborative spirit towards non-overlapping efforts, directing funding resources towards the boosting of skeletal genetic discovery.

Furthermore, modeling of human disease does not only require the standardization of both cellular and animal phenotyping (42), but oftentimes also rethinking of the disease definition. Employing better defined heritable traits (endophenotypes) would benefit both the etiological understanding of the disease in a particular patient, improve the targeted therapeutic approach with fewer side effects, and provide more effective treatments. Moreover, gender-balanced data are needed as most of the -omics studies to date were focused on women.

As we move closer towards an integrative multi-omics and holistic approach to skeletal diseases, consortia such as GEMSTONE become a fundamental tenet of modern genomics. In the long term, these efforts will allow meaningful and physiologically relevant data to be extrapolated, will allow identification of molecular diagnostic biomarkers and translation of findings into new therapeutic targets with higher effectiveness and fewer adverse effects, which will contribute to a higher quality of care for human skeletal diseases.

## Author Contributions 

MR, FR and DK initiated and organised the manuscript. EK, VP, BBa, NAL, IS and KS generated the figures for the manuscript. EK, VP, BBa, SR, JM and DK generated the tables for the manuscript. All authors contributed to the writing and approved the final manuscript.

## Funding

Funding was obtained from the GEMSTONE COST Action (CA18139).

## Conflict of Interest

The authors declare that the research was conducted in the absence of any commercial or financial relationships that could be construed as a potential conflict of interest.

## Publisher’s Note

All claims expressed in this article are solely those of the authors and do not necessarily represent those of their affiliated organizations, or those of the publisher, the editors and the reviewers. Any product that may be evaluated in this article, or claim that may be made by its manufacturer, is not guaranteed or endorsed by the publisher.

## Abbreviations

ATAC-Seq, Assay for Transposase-Accessible Chromatin using sequencing; bALP, bone alkaline phosphatase; BMD, Bone mineral density; BMSC, bone marrow mesenchymal stromal cells; circRNA, circular RNA; CTX, C-terminal telopeptide of collagen type I; DXA, Dual X-ray absorptiometry; eBMD, estimated bone mineral density; ENU, N-ethyl-N-nitrosourea; eQTL, Expression quantitative trait locus; GEO, Gene Expression Omnibus; GWAS, Genome-wide association studies; HGMD, Human Gene Mutation Database; Hi-C, high‐throughput chromosome conformation capture; KP, known pathogenic; lncRNA, long non-coding RNA; Mb, Mega base pairs; miRNA, microRNA; miR-SNPs, polymorphisms in miRNA genes; miR-TS-SNPs, SNPs that occur in the miRNA target site; mQTLs, DNA methylation quantitative trait locus; mRNA, messenger RNA; MSC, Mesenchymal stromal cells; MSK, musculoskeletal; ncRNA, non-coding RNA; NMR, nuclear magnetic resonance; NTX, N-terminal telopeptide of collagen type I; OoC, Organ-on-Chip; OI, Osteogenesis imperfecta; PBMC, peripheral blood mononuclear cell; PheWAS, Phenome-wide association study; PINP, procollagen type I N-terminal propeptide; pQTL, protein expression quantitative trait locus; QCT, Quantitative computed tomography; QTL, quantitative trait locus; RNA-seq, RNA sequencing; scRNA-seq, single cell RNA sequencing; SINE, short interspersed nuclear element; SNP, single nucleotide polymorphism; SRA, Sequence Read Archive; TAD, topologically associating domain; TF, transcription factor; TFBS, transcription factor binding site; TGS, third generation sequencing; TRAcP (TRAP), tartrate resistant acid phosphatase; UKBB, UK BioBank; WES, Whole exome sequencing; WGS, Whole genome sequencing.

## Glossary

**Table:**

ATAC-Seq	Assay for Transposase-Accessible Chromatin using sequencing
bALP	bone alkaline phosphatase
BMD	Bone mineral density
BMSC	bone marrow mesenchymal stromal cells
circRNA	circular RNA
CTX	C-terminal telopeptide of collagen type I
DXA	Dual X-ray absorptiometry
eBMD	estimated bone mineral density
ENU	N-ethyl-N-nitrosourea
eQTL	Expression quantitative trait locus
GEO	Gene Expression Omnibus
GWAS	Genome-wide association studies
HGMD	Human Gene Mutation Database
Hi-C	high‐throughput chromosome conformation capture
KP	known pathogenic
lncRNA	long non-coding RNA
Mb	Mega base pairs
miRNA	microRNA
miR-SNPs	polymorphisms in miRNA genes
miR-TS-SNPs	SNPs that occur in the miRNA target site
mQTLs	DNA methylation quantitative trait locus
mRNA	messenger RNA
MSC	Mesenchymal stromal cells
MSK	musculoskeletal
ncRNA	non-coding RNA
NMR	nuclear magnetic resonance
NTX	N-terminal telopeptide of collagen type I
OoC	Organ-on-Chip
OI	Osteogenesis imperfecta
PBMC	peripheral blood mononuclear cell
PheWAS	Phenome-wide association study
PINP	procollagen type I N-terminal propeptide
pQTL	protein expression quantitative trait locus
QCT	Quantitative computed tomography
QTL	quantitative trait locus
RNA-seq	RNA sequencing
scRNA-seq	single cell RNA sequencing
SINE	short interspersed nuclear element
SNP	single nucleotide polymorphism
SRA	Sequence Read Archive
TAD	topologically associating domain
TF	transcription factor
TFBS	transcription factor binding site
TGS	third generation sequencing
TRAcP (TRAP)	tartrate resistant acid phosphatase
UKBB	UK BioBank
WES	Whole exome sequencing
WGS	Whole genome sequencing
